# A distributable German clinical corpus containing cardiovascular clinical routine doctor’s letters

**DOI:** 10.1038/s41597-023-02128-9

**Published:** 2023-04-14

**Authors:** Phillip Richter-Pechanski, Philipp Wiesenbach, Dominic M. Schwab, Christina Kiriakou, Mingyang He, Michael M. Allers, Anna S. Tiefenbacher, Nicola Kunz, Anna Martynova, Noemie Spiller, Julian Mierisch, Florian Borchert, Charlotte Schwind, Norbert Frey, Christoph Dieterich, Nicolas A. Geis

**Affiliations:** 1 Section of Bioinformatics and Systems Cardiology, Klaus Tschira Institute for Integrative Computational Cardiology, Heidelberg, DE Germany; 2 Department of Internal Medicine III, University Hospital Heidelberg, Heidelberg, DE Germany; 3 German Center for Cardiovascular Research (DZHK) - Partner site Heidelberg/Mannheim, Heidelberg, DE Germany; 4 Informatics for Life, Heidelberg, DE Germany; 5 Digital Health Center, Hasso Plattner Institute, University of Potsdam, Potsdam, DE Germany

**Keywords:** Health care, Medical research

## Abstract

We present CARDIO:DE, the first freely available and distributable large German clinical corpus from the cardiovascular domain. CARDIO:DE encompasses 500 clinical routine German doctor’s letters from Heidelberg University Hospital, which were manually annotated. Our prospective study design complies well with current data protection regulations and allows us to keep the original structure of clinical documents consistent. In order to ease access to our corpus, we manually de-identified all letters. To enable various information extraction tasks the temporal information in the documents was preserved. We added two high-quality manual annotation layers to CARDIO:DE, (1) medication information and (2) CDA-compliant section classes. To the best of our knowledge, CARDIO:DE is the first freely available and distributable German clinical corpus in the cardiovascular domain. In summary, our corpus offers unique opportunities for collaborative and reproducible research on natural language processing models for German clinical texts.

## Background & Summary

Despite sustained declines in cardiovascular disease (CVD) mortality in many countries across Europe, CVDs still account for approx. 4.1 million deaths within European Society of Cardiology (ESC) member countries and have remained the most common cause of death within this region (45 and 39% of all deaths in females and males, respectively). Moreover, the prevalence of CVDs across Europe is still high with an estimated 113 million people living with CVD in the 57 ESC member countries, significantly contributing to patient morbidity and hospitalizations^1^.

At the same time, in clinical routine, large portions of data like patient anamnesis, cardiovascular risk factors and diagnosis continue to be stored in unstructured form, such as free text in doctor’s letters^2^. The predominantly hypothesis-driven strategies used in cardiovascular research should be complemented by computer-assisted methods. The comprehensive analyses of large clinical datasets using automatic information extraction methods will not only significantly expand the data sources for clinical care and research, but could also improve clinical decision-making and enable progress in personalized medicine^2^.

The rapid development in the field of natural language processing (NLP) in the past 15 years provided powerful tools for automatic text processing^3^. A high number of models, based on rule-based methods, statistical and more recently neural network methods were developed and validated for various tasks. While state-of-the-art (SOTA) supervised machine learning models require annotated data for training, all methods require annotated data for evaluation and quality control.

Therefore, shared corpora in the clinical domain are essential to support transparent and reproducible experiments and foster innovation in the field of clinical NLP^2,4,5^. In addition to their use for various medical information extraction tasks, e.g. they can be used for (1) training unsupervised or semi-supervised machine learning models, (2) the development of clustering or topic modelling methods^6^, (3) the development of pre-processing methods such as sentence-splitting, tokenization or part-of-speech tagging^7^, (4) domain adaptation of language models through pre-training techniques^8^, (5) the development of data augmentation techniques to overcome data scarcity^9^ or (6) use of the corpus as a reference corpus for collaborative cross-site clinical annotation projects.

Progress In clinical NLP can have a direct impact on clinical routine, as it enables clinicians to extract insights from large amounts of clinical textual data. For example: (1) to build clinical decision support systems that can be used to identify drug-drug interactions or adverse drug events or (2) to support clinical trial recruitment, by identifying patients based on their clinical anamnesis, diagnosis or demographics.

### State of research

Although the availability of textual data from medical domain has increased, there is still an immense need for high quality, but also freely accessible clinical text corpora. Particularly strict data protection regulations are a huge challenge that prevent publishing even small-scale data sets. Distributable English medical corpora in the U.S. must meet the regulations of the HIPAA (Health Insurance Portability and Accountability Act of 1996; https://www.hipaajournal.com/de-identification-protected-health-information/). Accordingly, the safe harbor section explicitly lists eighteen Protected Health Information (PHI) identifiers (e.g. person names, dates etc.), which need to be removed from a clinical document in order to define it as de-identified (https://www.hhs.gov/hipaa/for-professionals/privacy/special-topics/de-identification/index.html, accessed 10.09.2022).

Based on HIPAA several clinical text corpora in English were recently published. The MIMIC III (Medical Information Mart for Intensive Care) corpus^10^ is one of the most popular and largest data set. MIMIC III contains approximately 2 million free text notes from various clinical domains. In addition, annotated clinical corpora are published in context of shared tasks^11^, e.g. by the i2b2 foundation (>805,118 tokens, https://portal.dbmi.hms.harvard.edu/projects/n2c2-nlp), CLEF (http://clef-ehealth.org/), SemEval (http://alt.qcri.org/semeval2014/) and THYME^12^. The availability of these corpora led to a series of developments of information extraction frameworks in clinical setting^13^.

In contrast to the US HIPAA regulation, European data protection regulations, specifically the GDPR (General Data Protection Regulation), do not explicitly define how to de-identify a clinical document when sharing it (For a more detailed comparison between GDPR and HIPAA, see: https://iapp.org/news/a/gdpr-match-up-the-health-insurance-portability-and-accountability-act/, accessed 10.09.2022). However, lately, non-English European corpora were published under the GDPR, e.g. (1) MERLOT, a French clinical routine corpus containing 500 manually de-identified documents including 148,476 tokens^14^ or (2) IULA, a Spanish clinical record corpus, containing de-identified and shuffled sentences extracted from 300 clinical reports^15^. Unfortunately, to date there are only a handful of German medical corpora publicly available. This led to the development of German medical corpora containing medical guidelines or synthetically generated clinical texts^11,16–19^. The largest German medical corpus is currently GGPONC 2.0, containing approximately 1,8 million tokens, which builds upon clinical guidelines from the oncology domain^11^.

The only distributable German clinical routine corpus available contains 200 oncological discharge summaries (89,942 tokens, created between 2013–2016) from the university hospitals Berlin (Charité) and Tübingen^20^. As these data were collected retrospectively, each document needed to be carefully manually de-identified. Furthermore, all sentences in the corpus were shuffled in order to meet legal requirements of the clinical data protection officers and the ethics committee. Thus, only sentence-level medical information extraction (MIE) is possible requiring proper sentence segmentations, which is quite challenging in clinical texts. To the best of our knowledge, CARDIO:DE is the first freely available and distributable German corpus containing coherent documents from the cardiovascular clinical routine.

### Goals

The goal of CARDIO:DE is to foster NLP research in the German-speaking cardiovascular domain by publishing a freely available corpus containing cardiovascular doctor’s letters from clinical routine. To achieve this goal, we prospectively collected 500 doctor’s letters from the cardiology department at Heidelberg University Hospital. In addition, we publish two manually annotated layers:

1. for medication information extraction and
2. for section classification.

Furthermore, we present results of various baseline classifiers, trained and evaluated on both annotation layers.

### Corpus characteristics

**Fig. 1:**
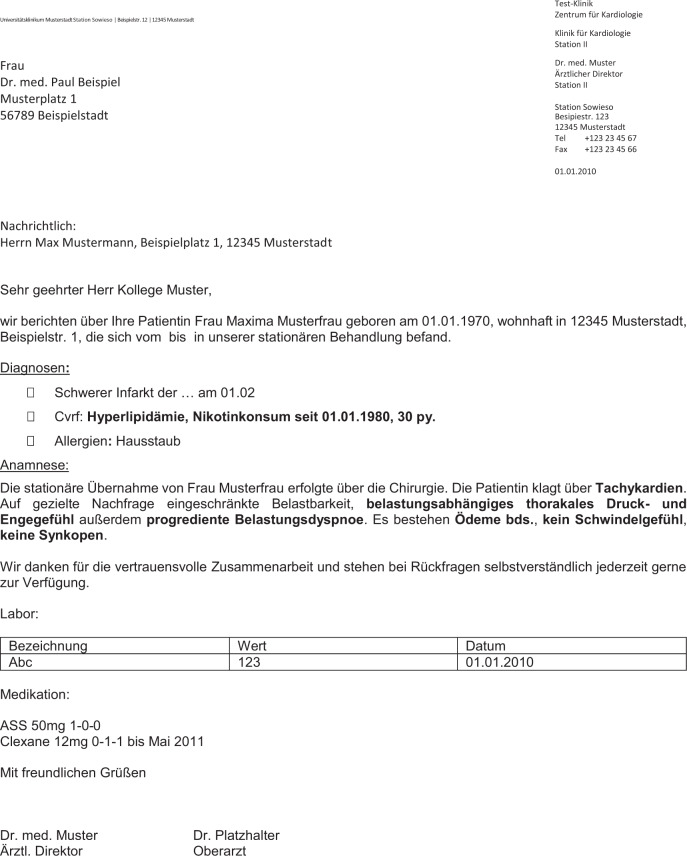
German dummy doctor’s letter from cardiology domain used in CARDIO:DE corpus. The letters are semi-structured binary MS-doc files. Most of the letters contain at least a header with contact information, a salutation, a diagnosis section, an anamnesis, laboratory values, medication plan and a conclusion/epicrisis.

CARDIO:DE encompasses 500 cardiovascular doctor’s letters covering a broad clinical spectrum of a tertiary care cardiovascular centre between 2020 and 2021. Our corpus covers 311 in-patient, 172 out-patient and 17 letters of the cardiac emergency room (chest pain unit; CPU). Thus, the included doctor’s letters cover both complex multiple-day hospitalizations as well as brief out-patient presentations. This results in the deployment of a representative collection of clinical documents, covering common doctor’s letter sections (e.g. anamnesis, physical examination, instrumental diagnostics, laboratory results, epicrisis, medication) in varying degrees and details. Figure 1 illustrates an excerpt of a doctor’s letter including common section types. The complete corpus contains 993,143 tokens, with approximately 31,952 unique tokens.

We randomly split our corpus into two parts (similar to Kittner *et al*., 2021^20^). CARDIO:DE400 contains 400 documents, 805,617 tokens and 114,348 annotations. CARDIO:DE100 contains 100 documents, 187,526 tokens and 26,784 annotations. Both corpora will be published for scientific research purposes. Scientific research excludes processing the data for marketing purposes. We will only publish annotations of CARDIO:DE400, annotations of CARDIO:DE100 will be kept inhouse as held-out data for a shared task on various MIE tasks, which we want to organize in the future.

**Fig. 2:**
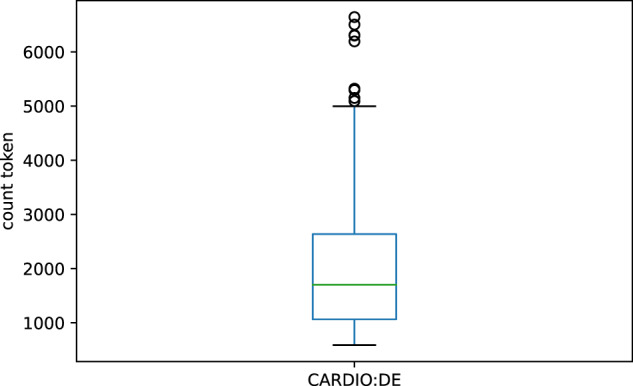
Token count per doctor’s letter. Distribution of token count per doctor’s letter in CARDIO:DE corpus. X-axis: token count.

Table 1 shows a quantitative analysis per CARDIO:DE splits. Figure 2 illustrates the quantitative analysis as a box plot for the whole CARDIO:DE corpus. In Table 2 we present the most common 50 whitespace separated token in CARDIO:DE including token count.

**Table 1:** Corpus token statistics.

	CARDIO:DE	CARDIO:DE400	CARDIO:DE100
Total	993,143	805,617	187,526
Mean	1,986	2,014	1,875
Min	588	588	597
25%	1,064	1,082	992
Median	1,704	1,764	1,448
75%	2,638	2,647	2,562
Max	6,644	6,644	5,322

Total token count and quantitative analysis of token count per doctor’s letter per CARDIO:DE split.

**Table 2:** Frequent token in CARDIO:DE.

Token	Count	Token	Count
Parameter	3,404	/nl	936
Datum (*date*)	3,388	links (*left*)	919
Wert (*value*)	3,381	Vorstellung (*presentation*)	913
Normb (*norm range*)	3,365	Beschwerden (*complaints*)	906
Dimension	3,365	Nachweis (*proof*)	899
min	1,999	rechts (*rechts*)	881
mmHg	1,843	Befund (*finding*)	872
empfehlen (recommend)	1,784	guter (*good*)	871
erfolgte (*took place*)	1,619	Ruhe-EKG (*resting ECG*)	858
Verlauf (*course*)	1,488	gute (*good*)	850
Patienten (*male patient*)	1,356	Medikation (*medication*)	846
wurde	1,336	Hinweis (*evidence*)	831
Mmol	1,285	Kontrolle (*check-up*)	828
Pumpfunktion (*pumping function*)	1,262	QRS	796
zeigte (*showed*)	1,175	Ödeme (*edema*)	788
Patientin (*female patient*)	1,139	QTc	786
Therapie (*therapy*)	1,120	gut (*good*)	773
Aufnahme (*admission*)	1,120	bitten (*asking for*)	756
gerne	1,108	Funktion (*function*)	755
ca.	1,057	leicht (*mild*)	754
Z.n	1,054	regelrecht (*correct*)	724
normal	1,026	LAD	723
unserer	1,018	regelmäßige (*regularly*)	720
Sinusrhythmus (*sinus rhythm*)	973	ASS	714
Risikofaktoren (*risk factors*)	937	Echokardiographie (*echocardiography*)	709

50 most common whitespace separated tokens in CARDIO:DE including count per token (selected English terms in brackets).

**Fig. 3:**
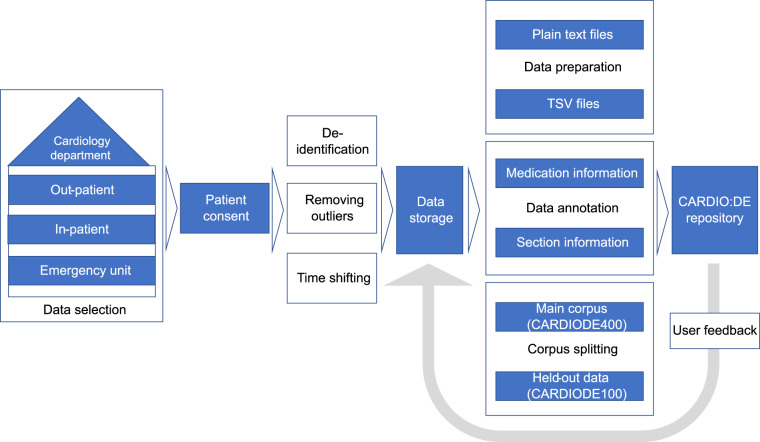
The CARDIO:DE development workflow. Visualization of the development process of CARDIO:DE. (1) Data selection, (2) Data collection and storage, (3) data preparation, annotation and corpus splitting, (4) CARDIO:DE repository.

We aim to publish cardiovascular doctor’s letters as close as possible to clinical routine documents. To achieve this, CARDIO:DE is based on a prospective study design with patient consent, which enabled us to keep the original document structure of clinical routine doctor’s letters (Fig. 3). While collecting patient consents can be time consuming and tedious, this procedure assures us to best comply with current data protection regulations in Germany. Moreover, similarly to recent corpus distribution projects^10,20^ we preserved the information on patient’s age and time/date in the documents. Thus, the corpus can be used for various information extraction tasks on document level in the cardiovascular domain. In a future version of CARDIO:DE, to further increase corpus consistency, all PHI placeholders will be replaced by semantic related surrogates, as proposed in Lohr *et al*., 2021^21^.

## Methods

### Ethics declarations

This study has been approved by the ethics committee of the Heidelberg University Hospital (S-498/2020) and has therefore been performed in accordance with the ethical standards laid down in the 1964 Declaration of Helsinki. All persons gave their informed consent prior to their inclusion in the study. The manuscript does not contain clinical studies or patient data.

### Data selection and collection

This study was designed monocentric, non-interventional, non-randomized, and prospective by collecting 500 patient consents between 2020 and 2021 in the cardiology department at the Heidelberg University Hospital. One doctor’s letter per patient was included into the CARDIO:DE corpus. Inclusion criteria were as follows: (1) age of at least 18 years (2) written consent signed by the patient; and (3) a diagnosis with a cardiovascular system disease. Patients were included after revision of the criteria by the recruiting study assistant, adequate information and subsequent signing of the CARDIO:DE consent form. By signing, the patient’s next generated doctor’s letter was included into the corpus. No study-specific additional examinations or further measures are performed within the scope of the project; thus the patient was not exposed to any additional risk.

We then exported each document and converted it from binary MS doc to MS docx to a plain text format (LibreOffice 6.1.5.2, https://wiki.documentfoundation.org/Faq/General/150, accessed: 22.08.2022) keeping the paragraph sections consistent, highlighted with the “¶” symbol in MS Word. We split each document by paragraph and tokenized each document using spaCy (v.3.2.1, language pipeline: de_dep_news_trf)^22^.

### De-Identification

All documents were initially de-identified using a deep learning model trained on manually annotated in-house data^23^. The model was trained on a pre-defined set of PHI classes. In a second step, clinical experts manually reviewed the automatic de-identified documents and replaced remaining un-deidentified PHI token with appropriate semantic placeholders. To keep the chronological order in the documents, we followed best-practice procedures^10^ by shifting all dates by a random number per document. Information about weekdays, time of day and seasons were kept. We also kept the information about patient age in the documents. If the patient was older than 80 years, we followed best-practice approaches^10^, by shifting age by a random number larger than 300.

We added three initial lines to each document containing pseudonymized meta information about: (1) admission date, (2) date of birth and (3) patient age. To further ensure anonymity, we removed outliers in laboratory values of each document, including patient height and weight. To identify outliers, we used a z-score approach^24^. Finally, we stored each doctor’s letter in our clinical data storage to save the corpus for further data preparation and annotation.

### Data annotation

We created two annotation layers to CARDIO:DE: (1) medication information on token-level and (2) information of section type on paragraph level. We used the annotation tool INCEpTION (v. 22.3) optimized for span annotations, including a monitoring and curation tool^25^. INCEpTION was installed as a web service in the clinical network to facilitate its use and data access for our annotation task force inside the clinical infrastructure.

### Annotation workflow

**Fig. 4:**
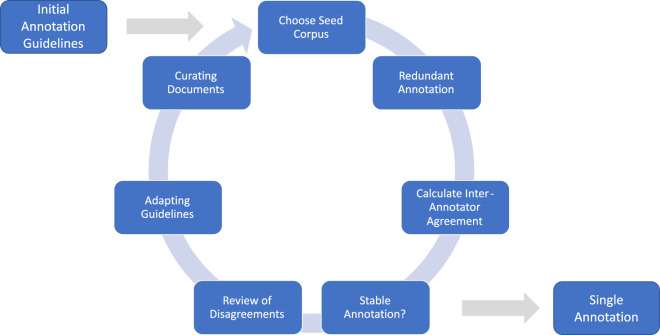
Annotation workflow. Iterative guideline adaptation process used for CARDIO:DE annotation layers. Redundant iterations helped synchronize the annotations with the guidelines. After meeting the IAA threshold, a final batch was assigned to each annotator.

We used well-established annotation methods^26–29^, including a guideline adaptation process by redundantly annotating documents involving an inter-annotator agreement score (IAA) in an iterative approach (Fig. 4).

After drafting initial guidelines with domain experts, a subset of documents was sampled from the main corpus for redundant annotation by all annotators. After each iteration the annotation master reviewed all annotations and documented all disagreements. To measure annotation quality an IAA score was calculated. During the following review meeting with all annotating participants including the annotation master all disagreements were discussed and a joint solution was defined. If necessary, the guidelines were adapted and a new iteration round was initialized until a pre-defined IAA threshold was met, depending on the annotation task. The annotation master curated all documents of each iteration based on the adapted guidelines. After redundant annotation was completed each annotator was assigned to a distinct subset of the remaining documents. To ensure high annotation quality nevertheless, the annotation master carefully reviewed all single annotated documents in compliance with the final guidelines.

### Medication information annotation

Our medication information annotation scheme is based on Uzuner *et al*., 2010^30^, and was adapted to the specific structure of our CARDIO:DE data (guidelines available as Supplementary File 1). As we performed a named entity recognition (NER) task, with many tokens not getting annotated with a class type, we used F1-score (harmonic mean between precision and recall) as IAA. We performed three iterations for redundant annotation with six annotators (four medical informatics master’s students, three with clinical experience and two medical students in their seventh semester (third clinical semester) with clinical routine experience) containing 15, 15 and 10 documents. The entire project lifetime, including preparation, annotation and evaluation, was three months. Approximate annotation time per document was 5–10 min.

Most of medication information in a doctor’s letter is listed in a separate semi-structured section (e.g. *Therapieempfehlung*, *Medikation bei Aufnahme*, …). In addition, we annotated medication information in narrative text sections. For all annotated medications in a doctor’s letter, the patient had to be the experiencer. We neither made any assumptions nor considered longitudinal information from external sources about a patient.

**Fig. 5:**
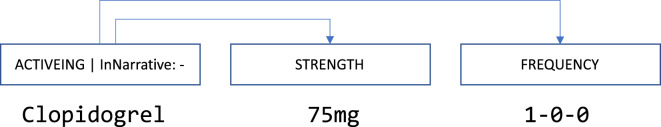
Example annotation for medication information. This annotated text snippet includes medication information annotations and relations to other tokens. The *ActiveIng* entity contains an attribute *inNarrative* to specify if the entity is inside a semi-structured section (−) or plain text section (+) of a doctor’s letter. In this example, the *ActiveIng* entity is inside a semi-structured section.

Our annotation objective was to identify a relevant drug (*Drug*) or active ingredient (*ActiveIng*) and its relation information (*Dosage*, *Route*, *Frequency*, *Duration*, *Strength*, *Reason* and *Form*). Moreover, we added a binary attribute (*inNarrative*) to each *Drug*/*ActiveIng*, to mark whether the medication information is in a semi-structured or in a plain text section (Fig. 5).

In this initial corpus version, we did not add entity normalization to the medication information layer.

### Section type annotation

Our section type annotation scheme is based on Lohr *et al*., 2018^31^, but is more coarse-grained and carried out on paragraph-level (guidelines available as Supplementary File 2). To measure the quality of annotations we calculated an IAA using Krippendorff’s alpha. Krippendorff’s alpha is a chance corrected inter-annotator agreement score and can be used for any number of annotators and class labels^32^. We performed three iterations for redundant annotation with three annotators (two clinical data scientists researching on clinical routine documents at the cardiology department, one research student assistant studying computational linguistics (B.A.) in sixth semester) containing 35, 30 and 20 documents. The project lifetime, including preparation, annotation and evaluation, was two months. Approximate annotation time per document was 3–8 min.

We annotated fourteen section types. Nine section types are mapped to HL7 CDA elements (Arztbrief Plus, v. 3.15, https://wiki.hl7.de/index.php?title=IG:Arztbrief_Plus, accessed 06.10.2022). Sections related to diagnosis are not mapped to CDA elements. The CDA standard separates diagnosis sections into *Entlassungsdiagnose (discharge diagnosis)* and *Aufnahmediagnose (admission diagnosis)*. Neither of them is explicitly part of doctor’s letters in CARDIO:DE. After consultation with cardiologists, we decided to use the most representative heading names in the original doctor’s letters as section class labels. There are typically two section headings related to diagnosis: (1) *AktuellDiagnosen*: This section contains discharge diagnosis information and is part of most of the letters. (2) *Diagnosen*: This section type contains admission or discharge diagnosis information. In the original documents in MS word format, important diagnosis information is commonly written as bold type. This information is not part of the documents in CARDIO:DE. After consultation with physicians, in addition to CDA section type *Befunde*, we annotated section types *KUBefunde* and *EchoBefunde*. Both appear frequently in CARDIO:DE letters and are considered relevant for cardiovascular clinical routine and research. Sections and paragraphs which cannot be mapped to one of the thirteen section types listed in Table 3 are annotated with the generic section type *Mix*.

**Table 3:** Section types in CARDIODE:

	CDA ID
Anrede (*Salutation*)	1.2.276.0.76.10.3001
AktuellDiagnosen (*Current diagnosis*)	—
Diagnosen (*Diagnosis*)	—
AllergienUnverträglichkeitenRisiken (*Allergies intolerances risks*)	1.2.276.0.76.10.3028
Anamnese (*History of present illness*)	1.2.276.0.76.10.3022
AufnahmeMedikation (*Admission medication*)	1.2.276.0.76.10.3029
KUBefunde (*Physical examination*)	—
Befunde (*Results*)	1.2.276.0.76.10.3100
EchoBefunde (*Echocardiographic findings*)	—
Labor (*LaboratoryResultObservation*)	1.2.276.0.76.10.4254
Zusammenfassung (*Hospital course*)	1.2.276.0.76.10.3021
Mix (*Other*)	—
EntlassMedikation (*Discharge medication*)	1.2.276.0.76.10.3031
Abschluss (*Final remarks*)	1.2.276.0.76.10.3034

CDA code for each section type, if available (English translation in brackets).

CARDIO:DE contains high-quality gold standard annotations for medication information and CDA compliant section types. Our IAA scores are comparable to previously published similar annotation projects (e.g. IAA from i2b2 corpus for medication information on token-level F1: 81.6–88%^33^, e.g. IAA for section types, median Krippendorff alpha: for seven classes: 84.6, 11–21 classes: 70–84.4^31^. Please note, that these IAAs are not completely comparable, due to different pre-processing steps and measurement procedures). Since the corpus is freely available for research purposes, excluding marketing purposes, we encourage the community to improve existing annotations and add new annotation layers to CARDIO:DE. Indeed, we think of CARDIO:DE as a facilitator and driver of collaborative NLP research in the German-speaking cardiovascular community. To support this goal, we also plan to organize shared tasks for various clinical NLP topics, such as concept normalization or negation detection. We will also be releasing new and updated annotation layers.

## Data Records

We publish CARDIO:DE for scientific research purposes and follow best-practice approaches of recently published clinical data sets^10,20^. Scientific research excludes processing the data for marketing purposes. The corpus contains detailed clinical care data of patients. In this context, CARDIO:DE needs to be used with care and respect^10^. We distribute the data via heiDATA (https://heidata.uni-heidelberg.de/). The corpus must be formally requested following instructions on the CARDIO:DE website in the Terms of Use section (10.11588/data/AFYQDY)^34^. This procedure also applies for the CARDIO:DE_EXP corpus, which includes additional experimental annotation layers (details, see Usage Notes section)^34^.

**Fig. 6:**
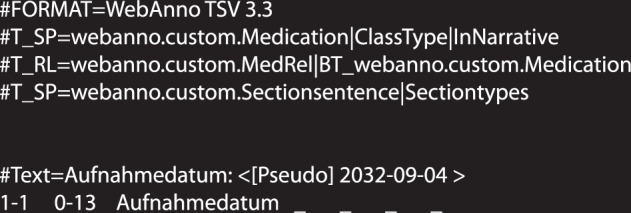
Structure of a CARDIO:DE400 tsv3 file. Excerpt of a tsv file from CARDIO:DE400_main corpus split. The header (initial four lines) contains meta information about the file structure: (1) version information, (2) medication information layer including attributes ClassType and InNarrative, (3) medication relation layer, (4) section information layer including attribute Sectiontypes. After the header each untokenized paragraph of the doctor’s letter is introduced by the key #Text. This is followed by a token-wise representation of the paragraph. Each line contains seven columns: (1) paragraph number and token number, (2) character offsets, (3) token, (4) medication information ClassType, (5) medication attribute InNarrative, (6) medication relation, (7) section information attribute Sectiontypes. CARDIO:DE100 tsv3 files do not contain annotation layer information in the header. Furthermore, the token-wise representation only contains the first three columns, excluding annotations columns. For further general details on the tsv3 format, see: https://inception-project.github.io/releases/24.3/docs/user-guide.html#sect_webannotsv.

CARDIO:DE (cardiode.zip) contains 500 doctor’s letters in plain text and in tsv3 format (WebAnno TSV version 3.3). The corpus is published as a zip file, containing all data files distributed in two folders (CARDIODE400_main, CARDIODE100_heldout). The tsv3 files of the CARDIO:DE400 split contain the untokenized and the tokenized format including annotations for the medication information and the section information layers. The tsv3 files of the distributed CARDIO:DE100 split only contain the untokenized and the tokenized format of each letter (Fig. 6).

CARDIO:DE_EXP (cardiode_exp.zip) is published with the same folder and file structure as CARDIO:DE. In addition to all annotations of CARDIO:DE, it contains three experimental medication information entity annotations: *Reason, Route and Dosage*.

## Technical Validation

### Annotation quality

#### Medication information

**Fig. 7:**
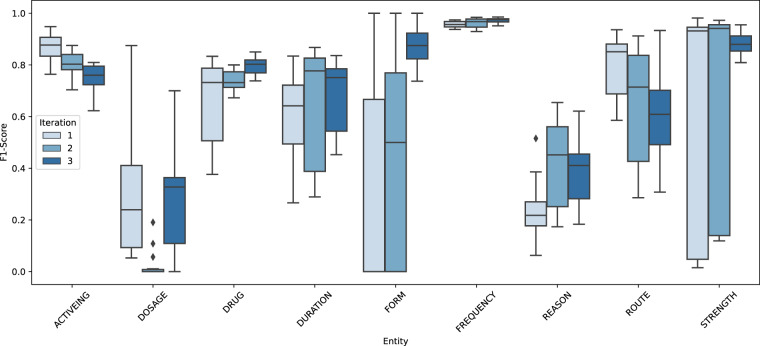
IAA medication information. Boxplot to illustrate the development of token level median IAA scores of all annotator combinations per iteration per medication information class (entity). X-axis: medication information class, y-axis: IAA F1-scores per iteration.

Figure 7 shows all token level median IAA scores of all annotator combinations per iteration per medication information class. Detailed information of IAA scores including standard deviation, see Table 4.

**Table 4:** IAA Medication information per class.

	Iteration 1	Stddev	Iteration 2	Stddev	Iteration 3	Stddev
ActiveIng	0.87	0.05	0.80	0.05	0.76	0.05
Dosage	0.24	0.28	0.00	0.05	0.33	0.19
Drug	0.73	0.16	0.73	0.04	0.80	0.03
Duration	0.64	0.16	0.78	0.22	0.75	0.13
Form	0.00	0.37	0.50	0.38	0.88	0.07
Frequency	0.96	0.01	0.97	0.02	0.98	0.01
Reason	0.21	0.11	0.45	0.17	0.41	0.12
Route	0.85	0.11	0.71	0.22	0.61	0.16
Strength	0.93	0.44	0.94	0.40	0.89	0.04

Median F1-scores per medication information class for all three redundant annotation iterations including standard deviation (stddev).

IAA could be improved consistently for three classes (*Drug*, *Form* and *Frequency*) over all iterations. For classes *Duration*, *Strength* and *Reason* IAA could be increased in second iteration and slightly decreased in third iteration. The complex *Reason* class still achieved a relatively low IAA (0.41) in iteration 3. For classes *Route* and *ActiveIng* IAA continuously decreased over all iterations. Standard deviation decreased in iteration 3 for all but the *Dosage* class, which showed the overall lowest IAA scores with a maximum of 0.33 in iteration 3.

Considering median micro average F1-score, token-wise F1-score could be improved in second iteration from 0.85 to 0.89, but only leveled at 0.85 in third iteration. Entity-wise median F1-score decreased in second iteration from 0.84 to 0.79, but increased to 0.81 in third iteration, which is 3 percentage points below IAA of first iteration (Table 5).

**Table 5:** IAA Medication information.

	Token-wise	Entity-wise	Relation annotation
Iteration 1	0.85	0.84	0.37
Iteration 2	0.89	0.79	0.62
Iteration 3	0.85	0.81	0.61

Median micro average F1 score for medication information (token-wise, entity-wise) and median micro-average F1-scores for medication relation annotations.

In addition to IAA for token level medication information, we calculated micro average F1-scores to measure the annotation quality of the annotations of medication information relation. We could improve IAA in second iteration by 0.25. In the last iteration IAA decreased to 0.61 (Table 5). As also reported by other publications, the IAA scores for relation annotation were generally lower, than for entity annotations^14,28^.

Overall, the synchronization of the annotation for medication information was very challenging. While annotation quality of medication information in the structured section of doctor’s letters quickly improved, annotation of more complex medication information samples in plain text remained difficult. Due to time restrictions, we stopped redundant annotations after the third iteration. Moreover, although some IAA scores could not be increased or even decreased, IAA scores of the most frequent classes (*Drug*, *ActiveIng*, *Strength*, *Form, Frequency*, *Duration*) achieved a sufficient quality (0.75–0.98).

One reason for the challenging synchronization might be rooted in the different educational backgrounds of the annotators. Generally, medical students and medical informatics students with experiences in clinical routine shared higher IAA scores. This was apparent for the *Reason* class, as this information demanded a more profound clinical knowledge. The *Dosage* class achieved lowest IAA scores as *Dosage* entities were easily confused as *Strength* entities (e.g. *max Zufuhr von 4 IE Insulin*/*h*: *4 IE* repeatedly annotated as *Dosage*; *Torasemid 5 mg 1-1-0*: *5 mg* repeatedly annotated as *Dosage*). In addition, *Strength* was occasionally annotated as *Dosage* in the structured medication sections. Overall *Dosage* entities are rarely represented in the corpus; thus, these results are not fully representative.

At the end of our annotation iterations, we had to face another challenge. To increase heterogeneity of the doctor’s letters in each iteration, we did not just randomly select letters from the complete corpus but restricted each sampling for each iteration to a specific time period of patient recruitment. For example; iteration 1 contained the first ten letters from the beginning of the projects recruiting phase, while iteration 2 contained letters from the middle of this phase. This resulted in a bias, as the patient recruitment was sometimes dominated by in-patients, out-patients or patients from the CPU. The structure of these letters can vary significantly, therefore the medication information in each iteration batch varied in its notational form. For future annotation projects, we recommend a more balanced distribution of such letters in each iteration in order to improve agreement between different iterations.

Due to the not satisfying IAA scores of *Reason, Route and Dosage*, we excluded these classes from CARDIO:DE. Still, to allow further improvement of annotation quality and to further experiment with these classes we distribute an experimental corpus named CARDIO:DE_EXP including all nine medication information classes, medication relations and section classes^34^.

**Fig. 8:**
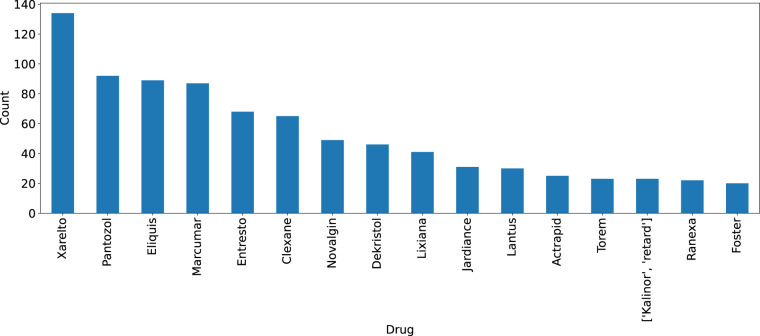
Drug entity counts. Counts of *Drug* entity annotations in CARDIO:DE (complete) corpus, if count > = 20 entities.

The final CARDIO:DE corpus contains in total 24,234 annotated medication information entities (Table 6) with 15,105 medication relations. The most frequent medication information classes are related to *ActiveIng*/*Drug* and their relations *Frequency* and *Strength*. Corpus statistics of CARDIODE_EXP are shown in Table 7. Figure 8 illustrates the most frequently annotated *Drug* entities in CARDIO:DE.

**Table 6:** Medication information statistics in CARDIO:DE.

Class	CARDIO:DE	CARDIO:DE400	CARDIO:DE100
ActiveIng	7,580	6,100	1,479
Frequency	6,491	5,150	1,341
Strength	6,367	5,026	1,341
Drug	2,093	1,680	414
Duration	1,527	1,233	294
Form	176	156	20
Total	24,234	19,345	4,889
inNarrative +/−	3,114/21,120	2,612/16,733	502/4,387

Entity counts (total and per medication information class) per CARDIO:DE split, including entity count in plain text (+) and in semi-structured sections (−).

**Table 7:** Medication information statistics in CARDIO:DE_EXP.

Class	CARDIO:DE_EXP	CARDIO:DE_EXP400	CARDIO:DE_EXP100
ActiveIng	7,580	6,100	1,479
Frequency	6,491	5,150	1,341
Strength	6,367	5,026	1,341
Drug	2,093	1,680	414
Duration	1,527	1,233	294
*Reason*	*1,498*	*1,224*	274
*Route*	633	514	119
*Dosage*	213	184	27
Form	176	156	20
Total	26,576	21,267	5,309
inNarrative +/−	3,254/23,322	2,728/18,539	526/4,783

Entity counts (total and per medication information class) per CARDIO:DE split, including entity count in plain text (+) and in semi-structured sections (−).

#### Section annotation

We could increase IAA from a Krippendorff’s alpha score of 0.91 to 0.96 (Table 8).

**Table 8:** IAA section types.

	Krippendorff Alpha	Stddev
Iteration 1	0.91	0.06
Iteration 2	0.89	0.17
Iteration 3	0.96	0.07

Median Krippendorff Alpha IAA score per iteration for the section annotation layer including standard deviation.

The final corpus contains in total 116,898 annotated paragraphs with section classes. The most frequent section class was *Labor* and *Befunde*. *Befunde* is a meta class, containing all kinds of findings, excluding *KUBefunde* and *EchoBefunde*. *Labor* contains laboratory information in a flattened tabular format. The least annotations are related to the class *Anrede*. This includes typically a single introductory sentence at the top of a doctor’s letter, containing information of the patient and the receiving department (Table 9).

**Table 9:** Section type statistics in CARDIO:DE.

Section type	CARDIO:DE	CARDIO:DE400	CARDIO:DE100
Anrede	501	402	99
AktuellDiagnosen	3,992	3,298	694
Diagnosen	5,769	4,725	1,044
AllergienUnverträglichkeitenRisiken	1,267	1,031	236
Anamnese	1,469	1,188	281
AufnahmeMedikation	2,651	2,058	593
KUBefunde	5,299	4,194	1,105
Befunde	12,155	9,636	2,519
EchoBefunde	1,856	1,566	290
Labor	67,640	55,420	12,220
Zusammenfassung	4,488	3,645	843
Mix	1,187	945	242
EntlassMedikation	5,124	4,090	1,034
Abschluss	3,500	2,805	695
Total	116,898	95,003	21,895

Section counts (total and per section type) per CARDIO:DE split.

During redundant section annotation, we faced a decrease in IAA in iteration 2. This was mainly due to a major change in the annotation scheme. Initially we annotated all lines of a section type with a span annotation. This resulted in slow performance of the INCEpTION tool, which made annotations unnecessarily laborious. The annotators decided to continue annotating just the first paragraph of a new section type. A new section type defines the end of the previous section type. The decision to only annotate the first paragraph of a new section type increased annotation speed significantly.

In iteration 2, the annotators missed some section ends because not every line had to be annotated anymore, especially with regard to section classes inside the *Befunde* class. Frequently annotators missed to mark the end of a *EchoBefunde* or *AllergienUnverträglichkeitenRisiken* section and as a result these sections covered too many paragraphs. After thorough review meetings, we were able to overcome this problem in Iteration 3.

Other issues concerned how to define the section type *EntlassMedikation*. Some doctor’s letters contained two explicit medication sections: section type *AufnahmeMedikation* at the beginning and section type *EntlassMedikation* at the end. *EntlassMedikation* was frequently introduced by the header ‘*Aktuelle Medikation’*. But a couple of letters only contained a single medication section at the beginning of a document. Therefore, some annotators interpreted these sections as *AufnahmeMedikation*, even though it is introduced with the header *‘Aktuelle Medikation’*. After review meetings and consultations with physicians, we defined this initial medication section with this header as *EntlassMedikation* in the guidelines.

## Usage Notes

### Data access

CARDIO:DE (and CARDIO:DE_EXP) must be formally requested via heiData following three steps:^34^

1. Sending a data request mail to heiDATA (data@uni-heidelberg.de) including a signed DUA form, addressing information about correct data usage and security standards. Under this licence, it is clearly prohibited, to identify individuals or try to contact or advertise them.
2. Including a group description and a project description (details, see below).
3. After a positive decision by the CARDIO:DE study director Christoph Dieterich (CARDIO:DE supervisor), the data requestor will receive detailed instructions how to download the corpus via heiDATA.

Data approval will require at least one week. The data request needs to contain the following information:

1. A signed DUA, signed by each data user individually.
2. A group description including the requestor’s (data user’s) name, affiliation, position and email address and website of the institution.
3. A project description of the research purpose (max. 150 words).
4. Name, affiliation, position, email address and signature of the responsible person to administer and manage the infrastructure on which CARDIO:DE will be stored.

The data request will be approved, if it contains all requested information and if it is in line with the data usage agreement (DUA). The data request will be rejected, if it contains incomplete or incorrect information or if it violates regulations of the DUA.

### Baseline classifier

We use baseline classifiers to demonstrate what current well-established and freely available machine learning models could achieve out-of-the-box on CARDIO:DE annotations. We trained our classifiers on annotations of CARDIO:DE400 and assessed their performance on annotations of CARDIO:DE100. For these models we performed neither hyperparameter tuning, nor architecture optimization. The results published here are intended to give a first impression of possible applications and how to train MIE models on CARDIO:DE annotations for both tasks. In a future shared task, researchers are invited to improve these baseline results.

### Medication information extraction

As an example use case, we evaluated a statistical and a neural machine learning model for medication information extraction. Our statistical model is based on a well-established conditional random field (CRF)^35^. The CRF was trained on basic linguistic features, including POS tags and context token. We used features proposed by Mikhail Korobov, see https://sklearn-crfsuite.readthedocs.io/en/latest/tutorial.html#features, for further details see Figure S1 in Supplementary File 3. Our neural model is based on a well-documented *Hugging Face* BERT language model for NER, pre-trained on different publicly available German language corpora (*deepset/gbert-base*)^36,37^.

In addition, we evaluated the freely available German GGPONC NER classifier (04_ggponc_fine_long), trained on SNOMED CT (https://confluence.ihtsdotools.org/display/DOCSTART/6.+SNOMED+CT+Concept+Model, accessed 08.09.2022) annotations in GGPONC 2.0^11^. The fine-grained scheme includes the entity type *Clinical Drug*. For a detailed description of this class, see GGPONC 2.0 guidelines: (https://github.com/hpi-dhc/ggponc_annotation/blob/master/annotation_guide/anno_guide.pdf, accessed 08.09.2022). *Clinical Drug* describes a pharmaceutical product produced for diagnostic or therapeutic purposes. While the guidelines do not exactly match our definition of *Drug/ActiveIng* we follow two mappings during evaluation, (1) mapping *Clinical Drug* to our *Drug/ActiveIng* (short) and (2) to *Drug/ActiveIng/Frequency/Strength (long)*.

**Fig. 9:**
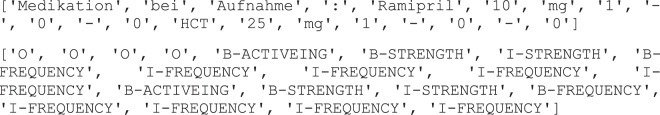
Training sample for medication information extraction. Example input and output sequences as Python lists of the medication information extraction task. The machine learning model predicts a medication information class for each of the input tokens using the IOB-scheme.

The task was designed as entity recognition classification. The objective of this task is to assign a set of six medication information classes (*ActiveIng*, *Drug*, *Duration*, *Form*, *Frequency*, *Strength*) to each input token. Medication information can consist of one single token or a sequence of tokens. Figure 9 shows a tokenized input snippet, containing 20 token and their assigned medication information classes. We evaluated this task using the F1-score, the harmonic mean between precision and recall per class, and the micro-average F1-score per classifier (Table 10). We evaluated token-wise and without an IOB scheme (short for inside, outside, beginning notation, see, https://en.wikipedia.org/wiki/Inside%E2%80%93outside%E2%80%93beginning_(tagging)), accessed 08.09.2022). Therefore, we removed all “B-” and “I-” substrings from the labels before calculating precision and recall. Token-wise results for precision, recall and F1-score of the GGPONC NER classifier are listed in Table 11.

**Table 10:** Token-wise precision (Pr), recall (Re) and F1-score (F1) results for medication information extraction per class and per model, including entity-wise F1-score in brackets and the micro average F1-score in the last row.

Class type	CRF			BERT			Count token (entities) in CARDIO:DE100
	Pr	Re	F1	Pr	Re	F1	
ActiveIng	0.84	0.83	0.83 (0.60)	0.80	0.91	**0.85** (0.86)	1,596 (1,479)
Drug	0.80	0.75	0.77 (0.77)	0.81	0.87	**0.84** (0.81)	532 (414)
Duration	0.80	0.73	0.77 (0.60)	0.78	0.89	**0.83** (0.59)	1,514 (294)
Form	0.47	0.33	0.39 (0.41)	0.57	0.71	**0.63** (0.60)	24 (20)
Frequency	0.97	0.97	0.96 (0.94)	0.96	0.98	**0.97** (0.94)	6,471 (1,341)
Strength	0.93	0.96	0.94 (0.92)	0.93	0.97	**0.95** (0.93)	2,692 (1,341)
Micro avg.	0.92	0.91	0.92 (0.87)	0.90	0.95	**0.93** (0.88)	12,829 (4,889)

For further information, hyper-parameters and, see Supplementary File 3.

**Table 11:** Token-wise precision, recall and F1-scores on CARDIO:DE100 for Clinical Drug class of GGPONC NER model.

Class type	Precision	Recall	F1-score
Drug (short)	0.13	0.67	0.21
Drug (long)	0.81	0.80	0.81

Considering token-wise and entity-wise micro average F1-score, BERT shows slightly higher results than CRF. Regarding token-wise F1-score BERT outperforms CRF over all classes. This is also true for the low-frequency class *Form*. Furthermore, this class achieved the overall lowest F1-score for both models.

Results of GGPONC NER shows the highest F1-score for the long mapping (81%), along with a balanced precision and recall score. The short mapping shows an overall much lower F1-score (0.21) along with a much lower precision (0.13) than recall score (0.67). For further evaluation results, see Supplementary File 4.

Considering different domains (oncology vs. cardiology) and document types (guidelines vs. doctor’s letters), the cross-domain GGPONC NER baseline showed impressive results on the CARDIO:DE100 corpus split. The short mapping achieved a recall of 0.67, while the precision score was only 0.13. This was especially due to issues while mapping our medication information classes based on our guidelines to the more generic GGPONC Snomed CT class *Clinical Drug*. We frequently observed, that GGPONC NER annotates token sequences such as 20 mg (*Strength*) or 1-0-0 (*Frequency*) as *Clinical Drug*, resulting in a high amount of false positives. Hence, our long mapping using the more comprehensive mapping of *Clinical Drug* to four classes *Drug*/*ActiveIng*/*Frequency*/*Strength* achieved both a high precision score (0.81) and a high recall score (0.80). These results show the potential of cross-domain models for German MIE. We therefore leave it to future work, to systematically evaluate the performance of publicly available German MIE models on already distributable German medical corpora. For a detailed analysis of GGPONC NER, see Supplementary File 4.

Current SOTA results for medication information extraction on English datasets achieve up to 0.95 F1-score^3^. Considering different data sets and hyperparameters a comparable German model for medication extraction for the classes *ActiveIng*/*Drug* (*Clinical Drug*) recognition in the GGPONC 2.0 corpus achieves 0.91 F-score, thus, outperforms our more fine-grained baseline distinguishing between *Drug* (0.81) and *ActiveIng* (0.86).

### Section classification

**Fig. 10:**
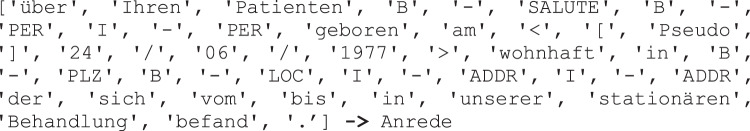
Training input for section classification. Example input paragraph as Python list including its section class of the section classification task. The machine learning model assigns a single section class to a given input paragraph.

Equal to the medication information extraction task, we evaluated a statistical and a neural model for section classification. For the statistical model we opted for a support vector machine (SVM)^38^. Our neural model is based on a well-documented *Hugging Face* BERT language model for sequence classification, pre-trained on different publicly available German language corpora (*deepset/gbert-base*)^36,37^. The objective of this task was to assign a set of fourteen section types (*Anrede, AktuellDiagnosen, Diagnosen, AllergienUnverträglichkeitenRisiken, Anamnese, AufnahmeMedikation, KUBefunde, Befunde, EchoBefunde, Labor, Zusammenfassung, Mix, EntlassMedikation, Abschluss*) to each input sample. An input sample consists of a paragraph of text (a paragraph is defined by the MS Word “¶” character) extracted from a doctor’s letter with no further context information. Figure 10 shows a tokenized example of an input sample, containing 48 tokens assigned to the *Anrede* class. We evaluated this task using the F1-score per class and the macro average F1-score per classifier (Table 12).

**Table 12:** Precision (Pr), recall (Re) and F1-score (F1) results per class and macro-average F1-score per model for section classification.

Section type	SVM			BERT			Count samples
	Pr	Re	F1	Pr	Re	F1	
Anrede	0.99	1.00	0.99	0.99	1.00	0.99	99
AktuellDiagnosen	0.73	0.51	0.60	0.67	0.67	**0.67**	694
Diagnosen	0.69	0.78	0.73	0.78	0.79	**0.79**	1,044
AllergienUnverträglichkeitenRisiken	0.97	0.94	0.95	0.97	0.96	**0.96**	236
Anamnese	0.90	0.81	0.85	0.81	0.93	**0.87**	281
AufnahmeMedikation	0.92	0.10	0.18	0.43	0.95	**0.59**	593
KUBefunde	0.98	0.97	0.98	0.98	0.97	0.98	1,105
Befunde	0.84	0.80	0.82	0.95	0.78	**0.86**	2,519
EchoBefunde	0.89	0.89	0.89	0.85	0.96	**0.90**	290
Labor	0.97	1.00	0.98	0.98	0.99	0.98	12,220
Zusammenfassung	0.90	0.95	0.92	0.94	0.94	**0.94**	843
Mix	0.87	0.64	0.74	0.65	0.88	**0.75**	242
EntlassMedikation	0.64	0.91	**0.75**	0.79	0.26	0.39	1,034
Abschluss	0.99	0.98	0.98	0.97	0.98	0.98	695
Macro avg. F1-score	0.88	0.80	0.81	0.84	0.86	**0.83**	21,895

For further information, confusion matrix and hyper-parameters, see Supplementary File 3.

Analyzing the macro average F1-score the BERT model outperforms the baseline by 0.02. Taking the per class F1-score into account, BERT achieves a better score in nine section classes. In four section classes both models achieve the same score, while for the class *EntlassMedikation* SVM achieves a higher F1-score.

Both models worst performing classes are related to medication sections. We observe a very low recall score for AufnahmeMedikation for the SVM and for EntlassMedikation for the BERT model. The SVM frequently classifies AufnahmeMedikation instances as EntlassMedikation. The BERT model, on the other hand, frequently misclassifies EntlassMedikation instances as AufnahmeMedikation, but to a lesser extent (confusion matrices for both models, see Figure S2 and Figure S3 in Supplementary File 3). Due to the fact that this task was performed only as a simple text classification task without further context information, these errors cannot be easily avoided, only by merging these class types or by adding context information to each input sample.

Regarding our baseline classifiers, considering different datasets, language and annotation guidelines, our final accuracy scores for SVM and BERT are comparable to similar published section classification results^31^.

Again, our two show cases on medical information extraction and section classification serve to demonstrate the great potential of this first German corpus from the cardiovascular domain. For future work, more sophisticated document segmentation methods need to be applied^39,40^.

## Supplementary information

Supplementary Material

## Data Availability

For information on software packages and versions used for pre-processing, see methods section. No additional custom code was implemented for CARDIO:DE.
