# Supplementary material for: A distributable German clinical corpus containing cardiovascular clinical routine doctor’s letters
Source: Sci Data. 2023 Apr 14;10:207. doi: 10.1038/s41597-023-02128-9 (PMC10104831; doi:10.1038/s41597-023-02128-9)
Supplement: Supplementary file 1 — Supplementary Material [file 41597_2023_2128_MOESM1_ESM.pdf]

Supplementary material of the manuscript “A distributable German clinical corpus containing cardiovascular clinical routine doctor’s letters”

Richter-Pechanski et al.
Manuscript Number: SDATA-22-01171

Content

1. Supplementary File 1 Annotation guidelines (Ver. 0.5) medication information - CARDIO:DE
2. Supplementary File 2 Annotation guidelines (Ver. 0.4) section annotation - CARDIO:DE
3. Supplementary File 3 Hyperparameters and evaluations CARDIO:DE baselines
4. Supplementary File 4 GGPONC NER evaluation on CARDIO:DE

Supplementary File 1 of the manuscript "CARDIO:DE A distributable German clinical corpus containing cardiovascular clinical routine doctor's letters"

Richter-Pechanski et al.
Manuscript Number: SDATA-22-01171

Annotations Guidelines (Ver. 0.5)

Medication Information - CARDIO:DE

Phillip Richter-Pechanski

May 18, 2022

1 Introduction

This document contains the fifth version of annotation guidelines for the CARDIO:DE corpus. The aim is to annotate 500 German discharge letters with medical information classes. The letters are already automatically (Richter-Pechanski et al. 2019) and manually de-identified.

Most of medication information of a patient are listed in semi-structured sections in a discharge letter, e.g. *Therapieempfehlung*, *Medikation bei Aufnahme*. However, we will annotate medication information described in other narrative sections, too.

For all of the listed medications, the patient must be the experiencer.

2 Preliminaries

Medication classes are annotated with the annotation tool INCEpTION. This project uses an iterative guidelines adaptation process using inter-annotator agreement, to ensure high annotation quality (Roberts et al. 2007). To easily track any changes to these guidelines a history section is provided in Appendix 1. Do not make assumptions or consider longitudinal information you may know about a patient in this workflow.

3 Classes to annotate medication information

For each medication listed in a discharge letter, the following information should be annotated, if available:

3.1 List of medication information classes

- **A**ctive Ingredient
- **D**osage
- **D**rug

- **D**uration
- **F**orm
- **F**requency
- **R**eason
- **R**oute
- **S**trength

4 Description of classes

Annotation objective is the identification of a relevant drug/active ingredient and its relation attributes (dosage, route, frequency, duration, strength and form).
In addition we annotate the DRUG/ACTIVEING class with the attribute TYPE, if the medication information is described in a narrative, non-semi-structured section. In this section we describe each medication information class, including an example.

1. ACTIVEING
   a) Attribute: TYPE: +/- Narrative.
   b) Attribute: TYPE: +/- Suggestion.
   c) A substance in a pharmaceutical drug that is responsible for the activity of a DRUG.
   d) Typically we annotate generic drug names, groups of active ingredients and expressions characterising general classes of drugs as ACTIVEING.https://www.overleaf.com/project/
   e) An abbreviation and a full name of an active ingredient are annotated with 2 separate annotations: *ASS <ACTIVEING> Acetylsalicylsäure <ACTIVEING>.*
   f) We also include brackets in an annotated entity: *Ass <ACTIVEING> -LRB- Acetylsalicylsäure -RRB- <ACTIVEING>.*
   g) If a DRUG is given with more than one generic name specified in brackets, we create one ACTIVEING annotation for all generic names: *Amiloretik <DRUG> -LRB- Amiloridhydrochlorid, Hydrochlorothiazid -RRB- <ACTIVEING>.*
   h) Conjunctions and other syntax as '/' or '+' that denote lists are not included in ACTIVEING annotation: *Vit. A <ACTIVEING> und Vit. E <ACTIVEING>*; *Salbutamol <ACTIVEING> und NaCl <ACTIVEING>.*
   i) If ACTIVEING is in brackets, as well including conjuctions, we annotate the whole bracket, including brackets: *-LRB- Amiloridhydrochlorid and Hydrochlorothiazid -RRB- <ACTIVEING>.*
   j) ACTIVEING examples:

- Generic drug names: *Wobenzym, Metoprolol, Acetylsalicylsäure, Salbutamol*
- Names of groups of active ingredients: *Antibiotikum, Vitamine, Endokarditisprophylaxe*
- Expressions characterising general classes of drugs: *Schmerztabletten, Schmerzmittel*
- *HCT*
- *Triple Therapie, duale Therapie*
- *Heimsauerstoff-Therapie*
- *Beta-Blocker-Therapie*
- *OAK*
- *antibiotische Therapie*
- *Bakterielle Endokarditisprophylaxe*
- *Lokalanästhesie*
- *Amiodaronaufsättigung*
- *Statin*

k) Non-ACTIVEING examples:

- We do not annotate a term if it does not denote a specific active ingredient: *Inhalation, salzarme Kost*
- *nephrotoxische Medikation*

2. DRUG

a) Attribute: TYPE: +/- Narrative.

b) Attribute: TYPE: +/- Suggestion.

c) Medication for which the patient is the experiencer.

d) A pharmacological substance that may or may not be approved for human use.

e) We typically annotate drug **brand names** with a DRUG annotation.

f) DRUG examples:

- Drug brand names: *DIAMOX, Belok-Zoc, Atacand, Aspirin*
- If a drug has a multi-word name that contains a dosage indication we annotate it as one DRUG entity. E.g. *Delix 5 plus*
- If a DRUG has a multi-word name that contains the corresponding ACTIVEING we annotate the whole chunk as DRUG. E.g. *Beloc zok -LRB-Metoprololsuccinat -RRB- mite <DRUG>*
- *Auto-CPAP-Therapie*

    - *Ultracain*
    - *Kalinor retard*

   g) Non-DRUG examples:
    - If there are two DRUGS (or ACTIVE INGREDIENTs) in one line, such that one of them does not come with any additional information (like dosage or frequency) we do not annotate the whole chunk. E.g.: *Fragmin P Forte 1-0-0 -LRB- bis Wiederansetzen von Aspirin -RRB-*
    - *Novalgin-Allergie*

3. DOSAGE
   a) The amount of a single medication prescribed.
   b) DOSAGE examples:
    - *halbe Tablette, 1-2 Hübe, 2x1 Hub*
    - However, if information about frequency and dosage are separated by a white space we create 2 annotations: *3 x 2 Hub -> 3 x <FREQUENCY> 2 Hub <DOSAGE>*
    - *100mg/d*

   c) Non-DOSAGE examples:
    - *erhöhen, abgesetzt, erhöhte Dosis.*
    - *Hochdosis*

4. DURATION
   a) Duration: how long the patient is expected to take the drug or has been taking the drug.
   b) DURATION examples:
    - *eine Woche lang, bis 14. März, für zunächst weitere 7 Tage, für 4 Woche nach Stentimplantation, Pause seit 14.03, Plavix 75 1-0-0 -LRB- nach 4 Wochen bitte absetzen -RRB-, ab dem 14.03, seit dem 14.03, am 03.02.2022, für 6 Monate*
    - *Aussetzen, abgesetzt*
    - *dauerhaft*
    - *Pause ab B-DATE*
    - *beginnend ab dem B-DATE*

5. FREQUENCY
   a) A schedule for drug administration that is defined by a doctor.
   b) Frequency implies how often each dose of the medication should be taken including both discrete and continuous values.

c) FREQUENCY examples:
   - *1x wöchentlich, 1x täglich, 1/Tag, 1-0-0, 2xtgl, bei Bedarf, 1x1 jeden 2. Tag, zur Nacht,z.N, tägl.*
   - Frequency may also contain dosage information *(2-0-2, 0-500-500, 10-10-10, 24 IE-0-0, Advagraf in mg 2-0-0)*
   - *times a day, etc.*
   - *specified time of day or hours*
   - *bei BEdarf, max 3x tägl.*
   - *bis einschließlich B-DATE*
   - *jeweils 30 bis 60 Minuten vor dem Eingriff*
   - *nach Bedarf/Bilanz*
   - *nach Schema*

6. STRENGTH
   a) The amount of active drug per unit, e.g. per ml, per ml.
   b) STRENGTH examples:
      - *25 mg*
      - Candesartan/HCT *8/12,5 V*itamin D 1000 IE
      - *4L/min*
      - *-LRB- Zieldosis: 10 mg/d -RRB-*
7. FORM
   a) A physical form of a drug prescribed by a doctor, such as solid, tablet, liquid, injectable, cream.
   b) FORM examples:
      - *Tablette, Flüssigkeit, Creme, Kautabletten, Kalinor Brause*
      - *Btl., Trp.*
8. REASON
   a) The medical reason for which the medication is stated to be given. Indications for which the medication would normally be given but which are not asserted by the text to be the reason for administering a medication are not included. Reasons/indications are usually given by adjective phrases or noun phrases. They usually correspond to diseases, signs or symptoms, and information related to other medications. The reason needs to be in the same sentence as ACTIVEING/DRUG or maximum in a preceding or succeeding sentence or list item. We include adjectives in annotations: *primärprophylaktischen Plaquestabilisierung.* A therapy, e.g. *antibiotische Therapie* is annotated as a

REASON, if it is further described by ACTIVEING. See, Fig. 14. Otherwise therypies are annotated as ACTIVEING.

b) REASON examples:

- Pantoprazol zum *Magenschutz*
- Zur *Stentverschlussprophylaxe* empfehlen wir eine ... mit ASS
- *NOAK*
- *Z.n. TAVI*
- *Kaliumsubstitution*
- *nach DES Implantation*

c) Non-REASON examples:

- *nach INR-Ziel 2,0-3,0*
- *akutem Nierenversagen* unter Aminoglycosiden.

9. ROUTE

a) A method for administering the medication (sublingual, intramusculär or i.m., intravenös or i.v.

b) ROUTE examples:

- Flutiform 250 *inh.*
- Heparin *Perfuso*
- *Inhalation* mit Sultanol und Atrovent
- *Fullface-Maske*
- *p.o.*, *i.v.*

The attributes inNarrative and isSuggested are only annotated for ACTIVEING and DRUG elements.

- inNarrative: the medication is located in a free text section of a discharge letters (including Diagnosis). The medication is not part of a semistrucured section e.g. *Medikation*, *Therapieempfehlung*, *Medikation bei Aufnahme ...*
- isSuggested: prescribtion of the medication is suggested, discussed or adviced. This has to be mentioned explicitly in the text with terms like *In Diskussion, wird in Erwägung gezogen* or similar. isSuggested is not used in semi-structured sections.

5 Special cases

- Articles: An indefinite article is not included in annotated entities. For example, in the noun phrase *ein Antibiotikum*, the span annotated is *Antibiotikum* not *ein Antibiotikum.*

- Prepositions: Avoid prepositions, e.g. *bei, mit*, except where they provide meaning or create a contiguous span. DO include when prepositional phrases add meaning such as in FREQUENCY and DURATION spans: E.g. *pro Tag, eine Woche lang, bis 14. März.*

- Punctuation: We include in annotation periods used with abbreviations: *1 Tabl.*.

- Brackets are included in annotations. E.g. *-LRB-Acetylsalicylsäure-RRB-* if the brackets contain only a single class. If there are > 1 distinct classes in a bracket, we exclude the bracket symbols. See Fig. 13.

- If brackets are not paired, we do not annotate them. E.g. *-LRB-Acetylsalicylsäure.*

- UNK (unknown token): If it is clear from the context what is meant with UNK we create a separate annotation for it: E.g. *UNK <DOSAGE> Tabl. <FORM>*. If UNK token is just placed after drug names, it is most probably a copyright symbol and we include this UNK character in a DRUG annotation: E.g. *MarcumarUNK <DRUG>, Kalium VerlaUNK <DRUG>.*

- We annotate composita containing a DRUG or ACTIVEING, e.g. Amiodaron-Ausättigung, Furosemidperfusor, Thrombozytenkonzentrat, Marcumar-Therapie.

- if FREQUENCY and STRENGTH including units as *mg, ml* or similar in one token combined, we choose STRENGTH. E.g. *2x5mg*, *400mg-200mg-200mg.*

- Splitting ROUTE and REASON: e.g. *orale* <REASON> Antikoagulation <ACTIVEING>.

- Further descriptions of DURATION are not annotated: e.g. *für 6 Monate* <DURATION> postinterventionell.

- The patient must be the experiencer of the medication. E.g. *Wenn eine Marcumar-Indikation vorliegt*, *Penicilin-Allergie*, *Methicillin*, *mit donorspezifischen Antikörpern*, *keine Retuximabtherapie* are not annotated.

- If DRUG name is including STRENGTH in middle of term, we annotate the while sequence of token as DRUG, see Fig. 8.

6 Referenzen

Richter-Pechanski, P., Amr, A., Katus, H. A., and Dieterich, C. (2019). Deep learning approaches outperform conventional strategies in de-identification of german medical reports. In GMDS, pages 101–109.

Roberts, A., Gaizauskas, R., Hepple, M., Davis, N., Demetriou, G., Guo, Y., ... Wheeldin, B. (2007). The CLEF corpus: semantic annotation of clinical text. In AMIA Annual Symposium Proceedings (Vol. 2007, p. 625). American Medical Informatics Association.

7 Upates

- 22.04.2022:
  - added attributes suggestion to ACTIVEING and DRUG class. Added description of attributed to section 4.
  - Added negative examples to REASON, DOSAGE.
  - Adding rules to annotate composita to section 5.
  - added positive examples for ROUTE, DRUG, ACTIVEING, FREQUENCY.
  - Restricted context window for REASON to max. succeeding or preceding sentence.
  - included *Sauerstoff* as ACTIVEING
- 05.05.2022:
  - Added rules to annotate ACTIVEING in context of conjunctions and brackets.
  - Added ACTIVEING example.
  - Split annotations of DRUG and ACTIVEING at conjunctions.
  - Annotation rules about context length of REASON.
  - including adjectives in REASON annotation.
  - isSuggested only used if suggestion is explicitly mentioned in section 4.
  - Added DURATION examples in section 4.
  - Dates, e.g. *am 02.03.2021* is DURATION.
  - if FREQUENCY and STRENGTH including units as *mg, ml* or similar in one token combined, we choose STRENGTH. E.g. *2x5mg*, added in section 5.
  - Adding REASON and ACTIVEING examples in section 4.
  - splitting REASON and ROUTE rules in section 5.
  - Excluding *postinterventionell* from DURATION.
  - Added Fig. 6-12.
  - Added REASON examples in section 4.
  - Added exception to not annotate allergies. Patient must be experiencer of medication, see section 5.
  - Added FREQUENCY examples.
  - Added DURATION example.
- 12.05.2022:
  - Added Fig 13, to visualize bracket annotation, if they contain more than one distinct class type.

  - Added example for STRENGTH.
  - Avoid prepositions in annotations, if they do not give medication related meaning, see section 5.
  - Exclude isSuggested attribute in semi-structured section. See, section 4.
  - Added rules to annotate therapies as REASON.

- 18.05.2022:
  - Corrected section 4, 1. g.) Amilorid to Amiloretik

8 Example snippets

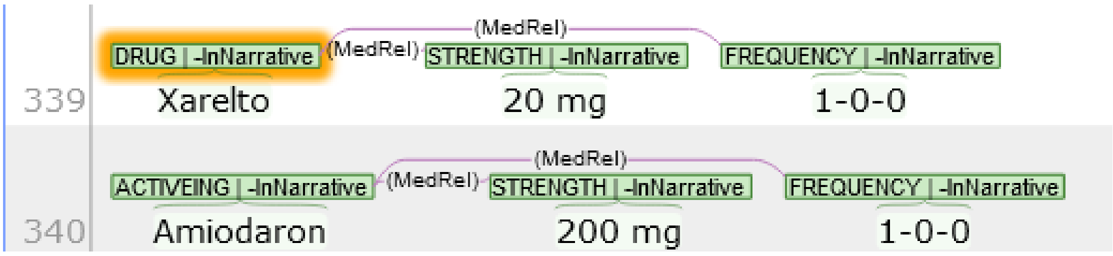

Figure 1: DRUG and ACTIVEING + additional information and relations.

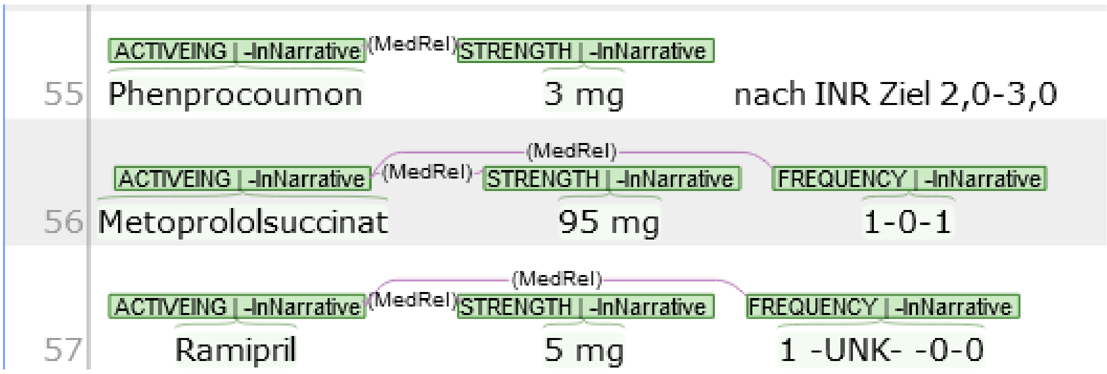

Figure 2: ACTIVEING including UNK token and relations.

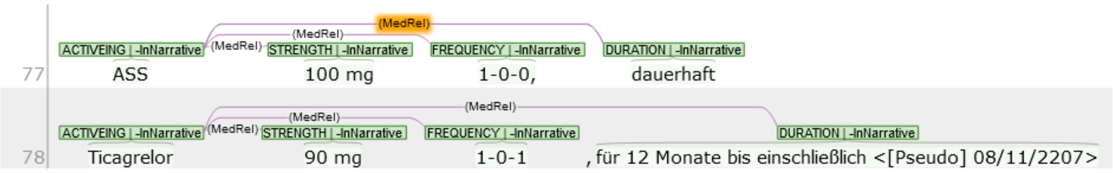

Figure 3: ACTIVEING including DURATION token and relations.

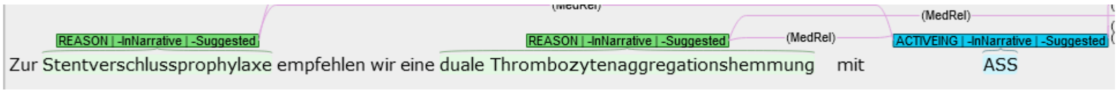

Figure 4: ACTIVEING including REASON, other information and relations in narrative text section.

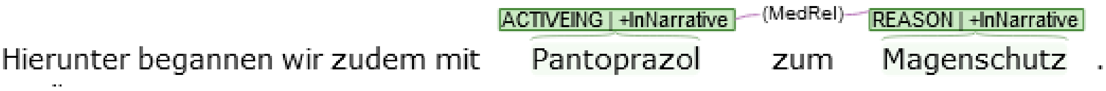

Figure 5: ACTIVEING including REASON, relations in narrative text section.

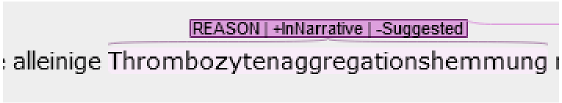

Figure 6: REASON, excluding *alleinige*, while including *duale*. See Fig. 4.

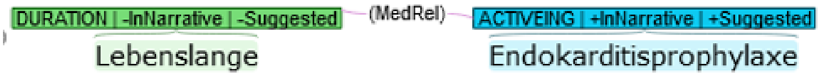

Figure 7: Example annotation DURATION and ACTIVEING

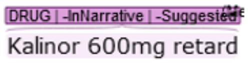

Figure 8: Special case DRUG including STRENGTH, annotated as one entity.

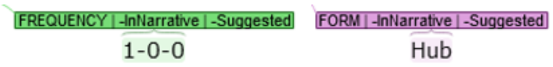

Figure 9: Hub is FORM, if not including a digit, as 3 Hübe.

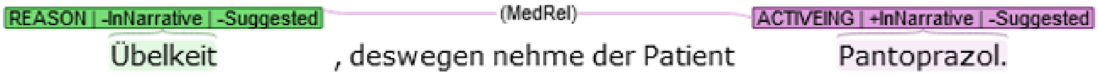

Figure 10: Example annotation REASON and ACTIVEING.

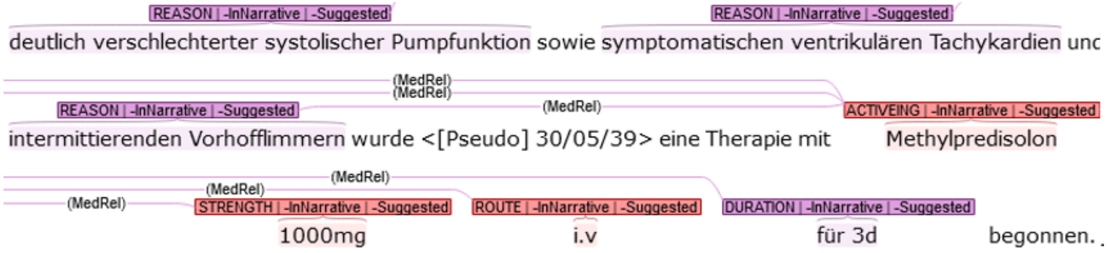

Figure 11: Example annotation, three separate REASONS.

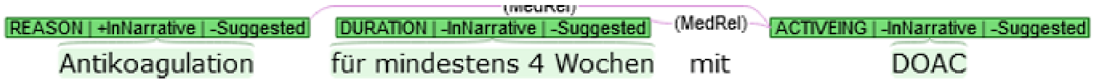

Figure 12: Example annotation, Antikoagulation and DOAC as REASON and ACTIVEING.

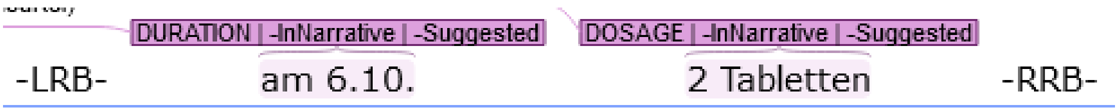

Figure 13: Example annotation, if a bracket contains more than one distinct class type. We exclude bracket signs in this case.

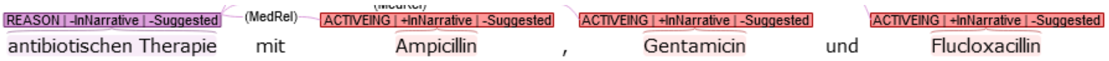

Figure 14: Example annotation, if a therapy is described by ACTIVEING, we annotate it as REASON.

Supplementary File 2 of the manuscript "CARDIO:DE A distributable German clinical corpus containing cardiovascular clinical routine doctor's letters"

Richter-Pechanski et al.
Manuscript Number: SDATA-22-01171

Annotation Guidelines (Ver. 0.4)

Section Annotation - CARDIO:DE

Phillip Richter-Pechanski

June 15, 2022

1 Introduction

This document contains the fourth version of annotation guidelines for the CARDIO:DE corpus. The aim is to annotate 500 German discharge letters with section classes. We follow section classes defined for the HL7-CDA (Clinical Document Architecture) `https://wiki.hl7.de/index.php?title=IG:Arztbrief_Plus`. The letters are already automatically (Richter-Pechanski et al. 2019) and manually de-identified.

2 Preliminaries

Section classes are annotated with the annotation tool INCEpTION. This project uses an iterative guidelines adaptation process using inter-annotator agreement, to ensure high annotation quality (Roberts et al. 2007). To easily track any changes to these guidelines a history section is provided in 7. Do not make assumptions or consider longitudinal information you may know about a patient in this workflow. We only annotate the first line of the section class. This can be a header, a simple token or a sequence of token. Examples, see 1, 2 and 3

3 Classes to annotate medication information

This section contains a list of all section types used in this project.

- **Ab**schluss
- **Ana**mnese
- **Anr**ede
- **AufnahmeD**iagnose
- **AufnahmeM**edikation

- **B**efunde
- **Ec**hoBefunde
- **EntlassD**iagnosen
- **EntlassM**edikation
- **K**uBBefunde (Körperlicher Untersuchungsbefund)
- **L**abor
- **M**ix
- **R**isikofaktorenAllergien
- **Z**usammenfassung

4 Description of classes

- **Ab**schluss
  - CDA code: `https://wiki.hl7.de/index.php?title=IG:Arztbrief_Plus#Abschlie.C3.9Fende_Bemerkungen`
  - Description: A free text formulated at the end of the letter corresponding to a final recommendation and greeting formula. Typically following medication or conclusion section at the end of a letter. Sometimes before laboratory values.
  - Example:
    * Selbstverständlich kännen Präperate mit gleichem Wirkstoff...
    * Mit freundlichen Grüßen
    * Ärztlicher Direktor Oberarzt
- **Ana**nmnese
  - CDA code: `https://wiki.hl7.de/index.php?title=IG:Arztbrief_Plus#Anamnesen`
  - Description: Current anamnesis. Typically initialized with section heading *Anamnese:*. We include spezielle Anamnese in this section class.
  - Example:
    * Anamnese:
    * Die Vorstellung erfolgte zur ...
    * Spezielle Krankheitsanamnese
- **Anr**ede

- CDA code: `https://wiki.hl7.de/index.php?title=IG:Arztbrief_Plus#Anrede`
- Description: This section contains the general introductory sentences of a discharge letter. They are summarized in one section and can contain salutations (e.g. "Dear colleague,..."), an initial naming of the patient possibly with the additional indication of the date of birth, etc.
- Example:
  * über Ihren Patienten ...

- **AufnahmeD**iagnose
  - CDA code: `https://wiki.hl7.de/index.php?title=IG:Arztbrief_Plus#Aufnahmediagnose`
  - Description: Specially labeled diagnosis made during the course of the admission examination. Typically initialized with *Diagnosen:*
  - Example:
    * Diagnosen:

- **AufnahmeM**edikation
  - CDA code: `https://wiki.hl7.de/index.php?title=IG:Arztbrief_Plus#Medikation_bei_Einweisung_.28Historie.29`
  - Description: Collected medication at the time of patient admission. *Medikation bei Aufnahme:*. Freqeuntly before anamnesis or risk factors.
  - Example:
    * Medikation bei Aufnahme: ...
    * Bisherige Medikation: ...

- **B**efunde
  - CDA code: `https://wiki.hl7.de/index.php?title=IG:Arztbrief_Plus#Befunde.2FErgebnisse`
  - Description: This section gathers all observation results/findings that have been collected for the patient. These may be echocardiography or electrocardiography findings, or findings from other imaging procedures. We do not include special section *Körperlicher Untersuchungsbefund* or laboratory values.
  - Example:
    * Lungenfunktionsdiagnostik: ...
    * 6-Minuten-Gehtest: ...
    * Ruhe-EKG: ...

- **Ec**hoBefunde

- CDA code: see, Befunde
- Description: This section gathers all observation regarding echogardiographie observations. It typically is initialized via heading *Echokardiographie* or *Transthorakale Echokardiographie* or similar.
- Example:
  * Echokardiographie vom <B-DATE>:
  * Transthorakale Echokardiographie ...
  * Dynamische Stress-Echokardiographie ...
  * TEE (Transösophageale Echokardiographie)

- **EntlassD**iagnosen
  - CDA code: `https://wiki.hl7.de/index.php?title=IG:Arztbrief_Plus#Entlassungsdiagnose`
  - Description: Diagnosis with which the patient was discharged. Typically initialized with *Aktuell:*
  - Example:
    * Aktuell: ...
- **EntlassM**edikation
  - CDA code: `https://wiki.hl7.de/index.php?title=IG:Arztbrief_Plus#Medikation_bei_Entlassung`
  - Description: Medication at discharge. Typically initialized with header *Therapieempfehlung*, *Medikation bei Entlassung* or similar.
  - Example:
    * Medikation bei Entlassung: ...
    * Medikation: ...
    * Bakterielle Endokarditisprophylaxe gemäß ...
- **K**uB (Körperlicher Untersuchungsbefund)
  - CDA code: see, Befunde
  - Description: Section containing general body measurements like height, weight, blood pressure. Not a distinct CDA section type, but part of Befunde. Typically initialized with *Körperlicher Untersuchungsbefund*, *KuB*, or similar.
  - Example:
    * KuB:
- **L**abor

  - CDA code: `https://wiki.hl7.de/index.php?title=IG:Arztbrief_Plus#Laborergebnis`
  - Description: Contains any laboratory values, typically in tabular format. Typically initilized with *Labor*, *Laborwerde vom ...* or a generic sentence like *Werte wurden ermittelt ...*
  - Example:
    * Labor
    * Werte wurden ermittelt zwischen dem ...

- **R**isikofaktorenAllergien
  - CDA code: `https://wiki.hl7.de/index.php?title=IG:Arztbrief_Plus#Allergien.2C_Unvertr.C3.A4glichkeiten.2C_Risiken`
  - Description: Information about allergies, intolerances and cardiovascular risks. Typically initialized with *Kardiovaskuläre Risikofaktoren*, *Cvrf*, *Allergien* or similar. In our discharge letters, risk factors and allergies are typically two distinct sections.
  - Example:
    * Cvrf: ...
    * Allergien: ...

- **Z**usammenfassung
  - CDA code: `https://wiki.hl7.de/index.php?title=IG:Arztbrief_Plus#Zusammenfassung_des_Aufenthalts`
  - Description: In the Epicrisis / Summary of stay section, a special summary review is recorded, an interpretation of the patient's events as well as the initiated therapy, which is intended for the physician providing further treatment. Typically initialized with *Epikrise*, *Zusammenfassung*, or similar.
  - Example:
    * Zusammenfassung: ...
    * Epikrise: ...

- *M*ix
  - CDA code: X
  - Description: All content, which is not fitting in one of the above sections classes. This excludes laboratory values, which we do not annotate. Can typically appear after risk factors, diagnosis, conclusion.
  - Example:
    * Externer Zuweiser: Strahlentherapie

    * Procedere: ...
    * Nächster Termin/Kontrolle ...
    * Reiseanamnese: ...
    * Soziales: ...
    * MRE: ...

5 Special cases

We do not annotate laboratory values, as they are typically inside improperly converted tables in our text documents.

6 Referenzen

Lohr C, Luther S, Matthies F, et al. CDA-Compliant Section Annotation of German-Language Discharge Summaries: Guideline Development, Annotation Campaign, Section Classification. AMIA Annu Symp Proc. 2018;2018:770-779. Published 2018 Dec 5.

7 Upates

- 08.06.2022
  - Removed class Familienanamnese
  - Added Labor class: as sub class of Befunde. CDA: `https://wiki.hl7.de/index.php?title=IG:Arztbrief_Plus#Laborergebnis`
  - Added examples for Echobefund class
  - Added examples to Mix class
  - Added examples to Anamnese, we include Spezielle Anamnese section to this classhttps://www.overleaf.com/project/603e2a6933b0630e7957ad4a
  - Added examples to Entlassmedikation
  - Specified annotation style in introduction. We only annotate first line of each section class. See 1, 2, 3.
- 14.06.2022
  - Define *Aktuelle Medikation* as Entlassmedikation
  - Labor section can start with irregular heading, like *Werte wurden ermittelt ....*
  - Risk factors can appear inside of *Befunde.*
  - Do not forget to switch section types, especially inside *Befunde* section.
  - Echo section can appear inside *Diagnosis* section.

  - Added example to *AufnahmeMedikation*.
  - Added example to *Echo*.

- 15.06.2022
  - Added examples to *Mix*.
  - The section introduced by the header *Aktuelle Medikation*, if located after anamnesis or risk factors, we annotate as *AufnahmeMedikation*.

8 Example snippets

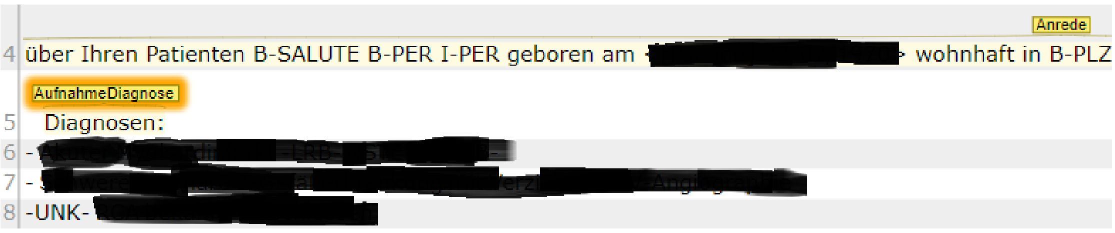

Figure 1: Example snippet to visualize section annotation style. We only annotate the first line of each section with the section class.

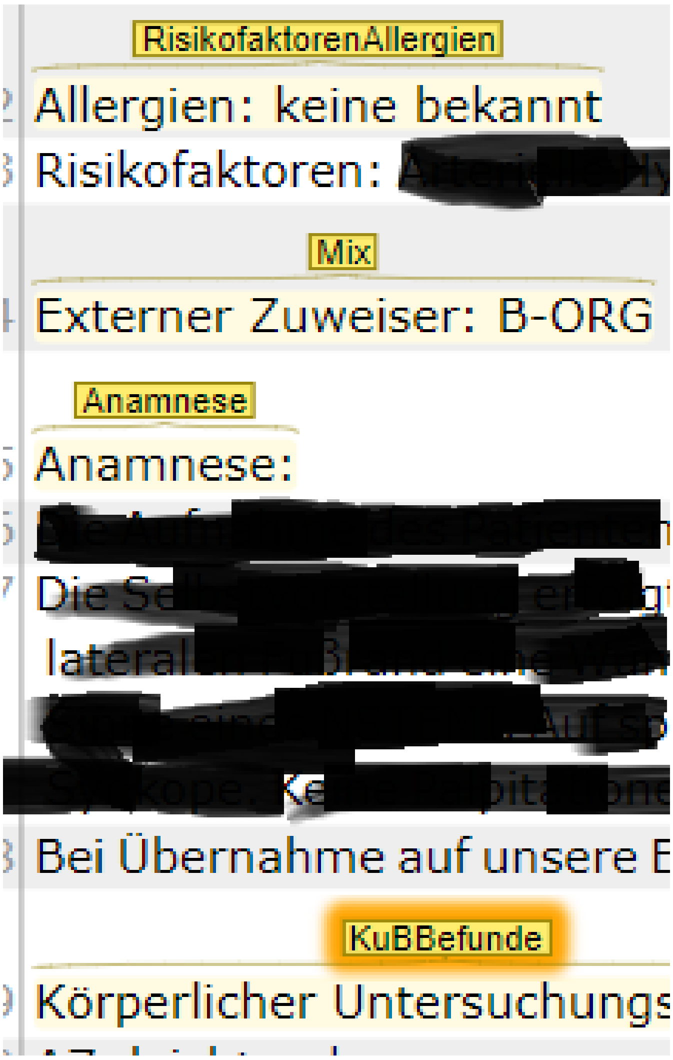

Figure 2: Example snippet two to visualize section annotation style. We only annotate the first line of each section with the section class.

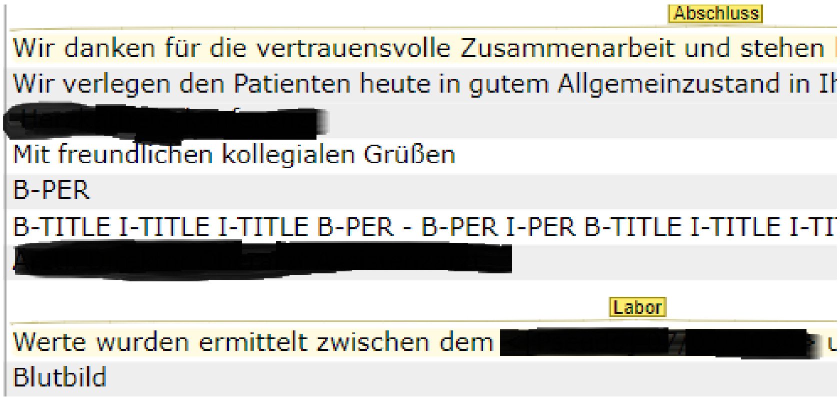

Figure 3: Example snippet three to visualize section annotation style. We only annotate the first line of each section with the section class.

Supplementary File 3 of the manuscript “ CARDIO:DE A distributable German clinical corpus containing cardiovascular clinical routine doctor’s letters”

Richter-Pechanski et al.

Manuscript Number: SDATA-22-01171

Table of contents

Medication information extraction

Table S1 BERT - Classification report (token-wise including B- and I- substrings)
Table S2 BERT - Classification report (token-wise removing B- and I- substrings)
Table S3 BERT - Classification report (entity-wise, strict IOB)
Table S4 BERT – Hyperparameters
Table S5 CRF - Classification report (token-wise including B- and I- substrings)
Table S6 CRF - Classification report (token-wise removing B- and I- substrings)
Table S7 CRF - Classification report (entity-wise, strict IOB)
Table S8 CRF - Hyperparameters
Figure S1 CRF– Features

Section classification

Figure S2 BERT– Confusion matrix.
Table S9 BERT - Features.
Figure S3 SVM– Confusion matrix.
Table S10 SVM– Hyperparameters and features.

Table S1 BERT for medication information extraction - classification report (token-wise including B- and I- substrings). Precision, recall, f1-score and support per medication class and micro/macro and weighted average scores.

	precision	recall	f1-score	support
B-ACTIVEING	0.83	0.92	0.87	1479
B-DRUG	0.83	0.90	0.86	414
B-DURATION	0.62	0.78	0.69	294
B-FORM	0.56	0.70	0.62	20
B-FREQUENCY	0.94	0.98	0.96	1341
B-STRENGTH	0.94	0.98	0.96	1341
I-ACTIVEING	0.50	0.61	0.55	117
I-DRUG	0.75	0.79	0.77	118
I-DURATION	0.79	0.88	0.83	1220
I-FORM	0.60	0.75	0.67	4
I-FREQUENCY	0.96	0.98	0.97	5130
I-STRENGTH	0.89	0.94	0.91	1351
micro avg	0.89	0.95	0.92	12829
macro avg	0.77	0.85	0.81	12829
weighted avg	0.90	0.95	0.92	12829

Table S2 BERT for medication information extraction - classification report (token-wise removing B- and I- substrings). Precision, recall, f1-score and support per medication class and micro/macro and weighted average scores.

	precision	recall	f1-score	support
ACTIVEING	0.80	0.91	0.85	1596
DRUG	0.81	0.87	0.84	532
DURATION	0.78	0.89	0.83	1514
FORM	0.57	0.71	0.63	24
FREQUENCY	0.96	0.98	0.97	6471
STRENGTH	0.93	0.97	0.95	2692
micro avg	0.90	0.95	0.93	12829
macro avg	0.81	0.89	0.84	12829
weighted avg	0.90	0.95	0.93	12829

Table S3 BERT for medication information extraction - Classification report (entity-wise, strict IOB). Precision, recall, f1-score and support per medication class and micro/macro and weighted average scores.

	precision	recall	f1-score	support
ACTIVEING	0.82	0.92	0.86	1479
DRUG	0.77	0.84	0.81	414
DURATION	0.51	0.68	0.59	295
FORM	0.52	0.70	0.60	20
FREQUENCY	0.92	0.96	0.94	1341
STRENGTH	0.91	0.96	0.93	1341
micro avg	0.84	0.92	0.88	4890
macro avg	0.74	0.84	0.79	4890
weighted avg	0.85	0.92	0.88	4890

Table S4 BERT for medication information extraction - Hyperparameters.

Parameter	Value
attention_probs_dropout_prob	0.10
classifier_dropout	null
hidden_act	gelu
hidden_dropout_prob	0.10
hidden_size	768.00
initializer_range	0.02
intermediate_size	3072.00
layer_norm_eps	1.00E-12
max_position_embeddings	512.00
model_type	bert
num_attention_heads	12.00
num_hidden_layers	12.00
pad_token_id	0
position_embedding_type	absolute
transformers_version	4.21.0
type_vocab_size	1
use_cache	true
vocab_size	30000
epochs	6
batch_size	64
learning_rate	5.00E-05
train_val_test_split	360/40 (CARDIO:DE400), 100 (CARDIO:DE100)
optimizer	AdamW

Table S5 CRF for medication information extraction - classification report (token-wise including B- and I- substrings). Precision, recall, f1-score and support per medication class and micro/macro and weighted average scores.

	precision	recall	f1-score	support
B-ACTIVEING	0.87	0.86	0.86	1479
B-DRUG	0.82	0.78	0.80	414
B-DURATION	0.68	0.62	0.65	294
B-FORM	0.50	0.35	0.41	20
B-FREQUENCY	0.95	0.97	0.96	1341
B-STRENGTH	0.95	0.95	0.95	1341
I-ACTIVEING	0.36	0.36	0.36	117
I-DRUG	0.72	0.60	0.65	118
I-DURATION	0.80	0.74	0.77	1220
I-FORM	0.33	0.25	0.29	4
I-FREQUENCY	0.97	0.97	0.97	5130
I-STRENGTH	0.88	0.94	0.91	1351
micro avg	0.91	0.91	0.91	12829
macro avg	0.74	0.70	0.71	12829
weighted avg	0.91	0.91	0.91	12829

Table S6 CRF for medication information extraction - classification report (token-wise removing B- and I- substrings). Precision, recall, f1-score and support per medication class and micro/macro and weighted average scores.

	precision	recall	f1-score	support
ACTIVEING	0.84	0.83	0.83	1596
DRUG	0.80	0.75	0.77	532
DURATION	0.80	0.73	0.77	1514
FORM	0.47	0.33	0.39	24
FREQUENCY	0.97	0.97	0.97	6471
STRENGTH	0.93	0.96	0.94	2692
micro avg	0.92	0.91	0.92	12829
macro avg	0.80	0.76	0.78	12829
weighted avg	0.92	0.91	0.91	12829

Table S7 CRF for medication information extraction - Classification report (entity-wise, strict IOB). Precision, recall, f1-score and support per medication class and micro/macro and weighted average scores.

	precision	recall	f1-score	support
ACTIVEING	0.86	0.85	0.86	1479
DRUG	0.79	0.75	0.77	414
DURATION	0.63	0.58	0.60	295
FORM	0.50	0.35	0.41	20
FREQUENCY	0.93	0.95	0.94	1341
STRENGTH	0.92	0.92	0.92	1341
micro avg	0.88	0.87	0.87	4890
macro avg	0.77	0.73	0.75	4890
weighted avg	0.88	0.87	0.87	4890

Table S8 CRF for medication information extraction - Hyperparameters.

Parameter	Value
algorithm	lbfgs
c1	0.1
c2	0.1
max_iterations	100
all_possible_transitions	False

Figure S1 CRF for medication information extraction - Features.

Features, as proposed by :
https://sklearn-crfsuite.readthedocs.io/en/latest/tutorial.html#features

```
def word2features(sent, i):
    word = sent[i][0]
    postag = sent[i][1]

    features = {
        'bias': 1.0,
        'word.lower()': word.lower(),
        'word[-3:]': word[-3:],
        'word[-2:]': word[-2:],
        'word.isupper()': word.isupper(),
        'word.istitle()': word.istitle(),
        'word.isdigit()': word.isdigit(),
        'postag': postag,
        'postag[:2]': postag[:2],
    }
    if i > 0:
        word1 = sent[i-1][0]
        postag1 = sent[i-1][1]
        features.update({
            '-1:word.lower()': word1.lower(),
            '-1:word.istitle()': word1.istitle(),
            '-1:word.isupper()': word1.isupper(),
            '-1:postag': postag1,
            '-1:postag[:2]': postag1[:2],
        })
    else:
        features['BOS'] = True

    if i < len(sent)-1:
        word1 = sent[i+1][0]
        postag1 = sent[i+1][1]
        features.update({
            '+1:word.lower()': word1.lower(),
            '+1:word.istitle()': word1.istitle(),
            '+1:word.isupper()': word1.isupper(),
            '+1:postag': postag1,
            '+1:postag[:2]': postag1[:2],
        })
    else:
        features['EOS'] = True

    return features
```

Figure S2 BERT for section classification – Confusion matrix.

	Abschluss	AktuellDiagnosen	AllergienUnverträglichkeitenRisiken	Anamnese	Anrede	AufnahmeMedikation	Befunde	Diagnosen	EchoBefunde	EntlassMedikation	KUBefunde	Labor	Mix	Zusammenfassung
Abschluss	683	0	0	0	1	0	0	0	0	0	0	0	1	10
AktuellDiagnosen	0	464	2	0	0	0	9	195	5	0	0	1	18	0
AllergienUnverträglichkeitenRisiken	0	0	226	0	0	0	3	2	0	0	0	1	4	0
Anamnese	0	0	0	261	0	2	4	0	6	0	0	2	0	6
Anrede	0	0	0	0	99	0	0	0	0	0	0	0	0	0
AufnahmeMedikation	0	0	0	0	0	563	4	0	0	25	0	1	0	0
Befunde	4	31	2	23	0	6	1974	35	36	4	19	291	70	24
Diagnosen	0	190	1	0	0	7	8	826	1	0	0	1	10	0
EchoBefunde	0	2	0	0	0	0	9	0	278	0	0	0	0	1
EntlassMedikation	6	4	0	0	0	731	13	0	0	270	0	5	5	0
KUBefunde	0	1	0	2	0	0	24	0	0	0	1076	2	0	0
Labor	0	0	0	0	0	2	25	0	0	41	0	12152	0	0
Mix	1	5	1	0	0	0	4	2	0	0	1	1	214	13
Zusammenfassung	8	0	2	35	0	0	0	0	1	0	0	0	7	790

Table S9 BERT for section classification - Features.

Parameter	Value
attention_probs_dropout_prob	0.1
classifier_dropout	null
hidden_act	gelu
hidden_dropout_prob	0.1
hidden_size	768
initializer_range	0.02
intermediate_size	3072
layer_norm_eps	1.00E-12
max_position_embeddings	512
model_type	bert
num_attention_heads	12
num_hidden_layers	12
pad_token_id	0
position_embedding_type	absolute
transformers_version	4.21.0
type_vocab_size	1
use_cache	true
vocab_size	30000
epochs	10
patience	1
batch_size	32
learning_rate	5.00E-05
train_val_test_split	360/40 (CARDIO:DE400), 100 (CARDIO:DE100)
optimizer	AdamW

Figure S3 SVM for section classification – Confusion matrix.

	Abschluss	AktuellDiagnosen	AllergienUnverträglichkeitenRisiken	Anamnese	Anrede	AufnahmeMedikation	Befunde	Diagnosen	EchoBefunde	EntlassMedikation	KUBefunde	Labor	Mix	Zusammenfassung
Abschluss	683	0	0	0	1	0	0	0	0	2	0	0	1	8
AktuellDiagnosen	0	355	1	0	0	0	55	264	6	2	0	3	3	5
AllergienUnverträglichkeitenRisiken	0	0	222	0	0	0	6	3	0	0	0	1	4	0
Anamnese	0	0	0	227	0	0	16	0	6	1	0	2	0	29
Anrede	0	0	0	0	99	0	0	0	0	0	0	0	0	0
AufnahmeMedikation	0	3	0	0	0	58	16	1	0	493	1	21	0	0
Befunde	1	18	0	17	0	0	2009	80	14	8	16	320	7	29
Diagnosen	0	98	4	1	0	1	94	815	3	10	0	18	0	0
EchoBefunde	0	3	0	0	0	0	22	8	257	0	0	0	0	0
EntlassMedikation	1	5	0	0	0	4	35	5	0	936	2	44	0	2
KUBefunde	0	1	0	1	0	0	29	1	0	0	1071	1	0	1
Labor	0	0	0	0	0	0	17	1	0	6	0	12196	0	0
Mix	0	4	0	0	0	0	62	2	0	1	0	3	156	14
Zusammenfassung	8	1	2	6	0	0	27	0	2	1	1	0	8	787

Table S10 SVM for section classification – Hyperparameters and features.

Parameter	Value
C	1
break_ties	False
cache_size	200
class_weight	None
coef0	0
decision_function_shape	ovr
degree	3
gamma	scale
kernel	rbf
max_iter	-1
probability	False
random_state	None
shrinking	True
tol	0.001
verbose	False
feature_vector_generation	TF-IDF vectorizer sci-kit (v. 1.0.2)
filter	german stopwords using nltk.corpus (3.7)

Supplementary File 4 of the manuscript "CARDIO:DE A distributable German clinical corpus containing cardiovascular clinical routine doctor's letters"

Richter-Pechanski et al.

Manuscript Number: SDATA-22-01171

Table of contents

Introduction 1

Evaluation 2

Introduction

GGPONC NER was trained on high-quality Snomed CT[1] annotations of the GGPONC 2.0 corpus (Borchert et al., 2022). The class *Clinical Drug* is a sub class of the Snomed CT class *Substance* and semantically overlaps with our *Drug/ActiveIng* class. However*, Clinical Drug* is more general and covers a broader range of medication information, including *Frequency and Strength*.

Table 1 Annotated snippet of a CARDIO:DE doctor's letter containing medication information showing CARDIO:DE gold standard annotations and the prediction of GGPONC NER

Token	CARDIO:DE Gold Annotations	GGPONC NER Predictions
Prävastatin	ACTIVEING	Clinical Drug
20	STRENGTH	Clinical Drug
0	FREQUENCY	Clinical Drug
-	FREQUENCY	Clinical Drug
0	FREQUENCY	Clinical Drug
-	FREQUENCY	Clinical Drug
1	FREQUENCY	Clinical Drug

As illustrated in Table 1 GGPONC NER annotates the whole text snippet with *Clinical Drug*. This indicates, that information like *Frequency* and *Strength* are part of *Clinical Drug*.[2] Therefore, we evaluated two types of mappings from our CARDIO:DE labels to *Clinical Drug* (Table 2).

Table 2 Mapping types from Snomed CT *Clinical Drug* to CARDIO:DE medication information classes.

Mapping type	GGPONC	CARDIO:DE
Short	Clinical Drug	Drug/ActiveIng
Long	Clinical Drug	Drug/ActiveIng/Frequency/Strength

[1] https://confluence.ihtsdotools.org/display/DOCSTART/6.+SNOMED+CT+Concept+Model, accessed 08.09.2022.
[2] For a detailed description of this class, see GGPONC 2.0 guidelines: https://github.com/hpi-dhc/ggponc_annotation/blob/master/annotation_guide/anno_guide.pdf, accessed 08.09.2022.

During class mapping, we removed the IOB format of all class labels.[3] The beginning and end of an entity are based on different assumptions due to different definitions of Clinical Drug and our labels, thus this scheme produced various annotation errors.

Evaluation

In our evaluations we renamed all mapped classes including *Clinical Drug* consistently to *DRUG*. GGPONC NER was released in four versions. We show the results of the best performing model on our data: *04_ggponc_fine_long* (Table 3-4).

Table 3: Results including precision, recall and F1-score for the DRUG class (short mapping, *04_ggponc_fine_long*).

	precision	recall	f1-score	support
DRUG	0.13	0.67	0.21	2128

Table 4: Results including precision, recall and F1-score for the DRUG class (long mapping, *04_ggponc_fine_long*).

	precision	recall	f1-score	support
DRUG	0.81	0.80	0.80	11291

Results of the short mapping show a low precision, while recall achieved 67%. This indicates a large amount of false positive predictions. The long mapping could clearly improve both precision and recall scores. In-depth analysis confirmed our assumptions, that the *Clinical Drug* class of Snomed CT covers our *Frequency* and *Strength* classes, too.

We further investigated frequently appearing false positive predictions of both models (Table 5-7).

Table 5: Laboratory values are annotated as Clinical Drug by GGPONC NER.

Token	CARDIO:DE Annotation	GGPONC NER Prediction
Albumin	O	Clinical Drug

Table 6: Technical devices are annotated as Clinical Drug by GGPONC NER. In GGPONC, cardiovascular devices are rarely mentioned. GGPONC NER recognizes this term as medical, but classifies it to the wrong class.

Token	CARDIO:DE Annotation	GGPONC NER Prediction
Septal	O	Clinical Drug
Occluder	O	Clinical Drug

Table 7: More generic text sequences about medication are annotated as Clinical Drug by GGPONC NER. The whole context in the doctor‘s letter is: "Selbstverständlich können auch preiswertere

[3] https://en.wikipedia.org/wiki/Inside%E2%80%93outside%E2%80%93beginning_(tagging)

wirkstoffgleiche Präparate anderer Hersteller verwendet werden“. In CARDIO:DE guidelines we only annotate medication information, where the patient is the experiencer. This might be due to the text type of GGPONC, guidelines. They often speak in very abstract terms about medication information.

Token	CARDIO:DE Annotation	GGPONC NER Prediction
Preiswertere	O	Clinical Drug
,	O	Clinical Drug
wirkstoffgleiche	O	Clinical Drug
Präparate	O	Clinical Drug

Frequent false negatives of both models were *ActiveIng* like: ASS, Clopidogrel or Vitamin D. In addition *Drugs* like Panzytrat or Tromcardin were frequently not recognized as *Drug*.

In Table 8-9 we show results of the *02_ggponc_fine_short* model. Details, see (Borchert et al., 2022).

Table 8: Precision, recall and f1-score for the DRUG class (short mapping, *02_ggponc_fine_short*).

	precision	recall	f1-score	support
DRUG	0.61	0.77	0.68	2128

Table 9: Precision, recall and f1-score for the DRUG class (long mapping, *02_ggponc_fine_short*).

	precision	recall	f1-score	support
DRUG	0.61	0.15	0.23	11291

Further investigations and experiments to compare both model types we leave for future work.
